# Vessel detection and classification from spaceborne optical images: A literature survey

**DOI:** 10.1016/j.rse.2017.12.033

**Published:** 2018-03-15

**Authors:** Urška Kanjir, Harm Greidanus, Krištof Oštir

**Affiliations:** a ZRC SAZU, Department of Remote Sensing, Novi trg 2, 1000 Ljubljana, Slovenia; b European Commission, Joint Research Centre (JRC), Directorate for Space, Security and Migration, Via E. Fermi 2749, I-21027 Ispra (VA), Italy; c Faculty of Civil and Geodetic Engineering, University of Ljubljana, Jamova cesta 2, 1000 Ljubljana, Slovenia

**Keywords:** Ship detection, Ship classification, Vessel detection, Vessel classification, Sea target detection, Object recognition, Optical satellite data, Maritime domain awareness

## Abstract

• A review of 119 papers on ship detection and classification from optical satellite.
• From 1978 to March 2017, showing an exponential growth in the number of papers.
• Most published methods have very limited validation.
• While big steps have been made, automatic algorithms are still far from perfect.
• Increase in new observation and processing capabilities promises rapid advances.

## Abstract (sl)

This paper provides an overview of existing literature on vessel/ship detection and classification from optical satellite imagery. Although SAR (Synthetic Aperture Radar) is still the leading technology for maritime monitoring, the number of studies based on optical satellite data is quickly growing. Altogether we analysed 119 papers on optical vessel detection and classification for the period from 1978 to March 2017. We start by introducing all the existing sensor systems for vessel detection, but subsequently focus only on optical imaging satellites. The article demonstrates the temporal development of optical satellite characteristics and connects this to the number and frequency of publications on vessel detection. After presenting the methods used for optical imagery-based vessel detection and classification in detail, along with the achieved detection accuracies, we also report possibilities for fusing optical data with other data sources. The studied papers show that the most common factors greatly influencing the vessel detection accuracy are the following: different weather conditions affecting sea surface characteristics, the quantity of clouds and haze, solar angle, and imaging sensor characteristics. All these factors bring great variations in the selection of the most suitable method; some still continue to pose unsolved challenges. For higher relevance and wider usage, we suggest that the algorithms for detection and classification should support a variety of targets and meteorological conditions, and ideally also a variety of optical satellite sensors. At least, they should be tested on many images under different conditions. This is not usually the case in the existent literature. We also observed that many authors omit an appropriate performance quantification, which is critical for a practical assessment and a numerical comparison of the presented algorithms. Overall it can be seen that vessel monitoring from spaceborne optical images is a popular research topic and has a great operational potential in the near future due to the large amount of satellite data, much of it free and open.

## 1 Introduction

Measuring and monitoring human activity at sea is a topic of major and increasing interest. From fishing, drilling, exploration or cargo transport, to carrying passengers or tourism, efforts are expended – by governmental as well as commercial actors – to remain aware of what is going on. Maritime Domain Awareness (MDA) has been defined as the effective understanding of any activity associated with the maritime domain that could impact upon the security, safety, economy, or environment (IMO, 2010); and the maritime domain has been defined as all areas and things of, on, under, relating to, adjacent to, or bordering on a sea, ocean, or other navigable waterway, including all maritime-related activities, infrastructure, people, cargo, and vessels and other conveyances (EU, 2008, US Government, 2004). To achieve proper MDA, a lot of information is needed. MDA is enabled by the combination of information from land, sea-based, airborne, and satellite sensor systems, augmented with heterogeneous information from geographical information systems (GIS) and vessel information repositories (Dekker et al., 2013). But in any case, knowledge of positions and behaviours of vessels is a cornerstone. Vessel detection has a wide range of applications, in the areas of maritime safety, marine traffic, marine pollution, maritime spatial planning, fisheries management, illegal fishing, defence and maritime security, maritime piracy, irregular migration, border control, etc. Ships can be easily discerned in optical images taken from space. Hence, there is merit in considering the contribution of ship detection from satellite optical images.

As to wording, within the literature two terms are widely used for man-made objects on the sea surface: ‘ship’ and ‘vessel’. Although the majority of authors use the term ‘ship’ in their research, the expression ‘vessel’ is more generic, covering a variety of sea targets, ranging from smaller to bigger objects, and including very small boats, canoes, etc., as well as uncommon objects such as floating docks. Both terms will be deployed throughout this paper without an intended difference in meaning.

Many systems are available that can contribute to collecting knowledge on the presence and activity of ships. Usually, a distinction is made between cooperative and non-cooperative systems. With the former, the ships communicate information about themselves. With the latter, observation systems are used to collect information without any cooperation from the ships.

In accordance with a number of global, regional and national regulations, particular classes of vessels have to be equipped with shipborne transponders that transmit the vessel's identity and position at certain repeat intervals. One of the most common tracking systems is the Automatic Identification System (AIS), which is designed to automatically provide continuous information on location to other vessels and to coastal authorities (IMO, 2000). AIS was originally designed for short-range operation, and beside it, Long-Range Identification and Tracking (LRIT) was established as an international system by IMO (IMO, 2006). As a third widely used automatic reporting system, the Vessel Monitoring System (VMS) is used by authorities to keep track of fishing fleets (FAO, 1998). Whereas the carriage requirements for AIS and LRIT are globally set by IMO for the medium and large ships (see the IMO references for details), VMS carriage is instead regulated nationally or regionally. For some other systems see e.g. JRC (2008). While these systems are powerful tools to track the participating vessels, they only give a partial picture of the situation. Most small (< 300 tons) vessels do not need to carry AIS or LRIT, and small or even all fishing vessels do not carry VMS depending on the region. In addition, it has been observed that illegally operating vessels turn off or even spoof their mandatory position reports. Furthermore, LRIT and VMS data have a restricted access as they are collected by specific government authorities on specific legal bases. Therefore, we cannot count on the cooperative systems to provide comprehensive maritime domain awareness.

Concerning (non-cooperative) observing systems, the main sensors for maritime surveillance are, apart from visual sighting, optical camera, infrared camera and radar. These can be deployed from shore, ship, aircraft or satellite. Each sensor type and each platform has its own strengths and drawbacks, related to characteristics such as spatial resolution, update rate, range, coverage, persistence, latency and cost. Satellite-based sensors have the specific advantages of remote access, global reach, regular update and high data collection volume, so that in some scenarios they are the only feasible option and in others the most economic one. The usage of satellite images is therefore an essential tool to find vessels on the sea. In particular, satellite-based *radar* images, usually as Synthetic Aperture Radar (SAR), have become popular for maritime surveillance: ships are detected relatively easily, and independently of the presence of clouds or daylight. But the interest in the potential of optical images for maritime surveillance has dramatically increased recently, maybe in the first place due to the increase in optical imaging satellites. Indeed, it will be shown later that both the number of optical satellites and the number of publications on ship detection in optical images have grown exponentially during the last decade. Under the EU's Copernicus program for Earth observation, data from the Sentinel-2 optical satellite are even collected routinely and provided free and open to all since December 2015 (EC, 2016). Therefore, a review on this topic seems timely.

From the point of view of image analysis, object detection has been one of the most popular research topics and challenging tasks in remote sensing science in the last decades. There are numerous examples of machine-assisted or fully automatic detection of (small) objects in the fields of geosciences, geography, planning, infrastructure control, engineering, nearshore fixed object mapping, and homeland security (Marshburn et al., 2009). Ship detection is a special case of (small) object detection, where the background has the particular characteristics of the sea surface (further discussed in Section 4.2.2). The demand for automated analytical methods for remote sensing data is driven by the plethora of existing Earth-orbiting sensors and their daily generation of terabytes of data with different spatial, spectral, radiometric and temporal resolution (Hay et al., 2005). The detection systems are therefore faced with the need to process massive amounts of incoming data, often with the requirement to react in near-real time (Bi et al., 2010). Automated real-time vessel detection is a key point to various maritime missions.

The word ‘detection’ is used in the literature sometimes in a wide and sometimes in a narrow sense. The complete vessel detection procedure refers to the detection in the wide sense, and is composed of three main sequential steps:

1. Vessel detection (in the narrow sense): finding vessel candidates in the image and locating them;
2. Vessel classification: discriminating detected targets between vessel/non-vessel and then getting the class of the vessel (e.g. fishing, tanker, cargo); and
3. Vessel identification: establishing the unique identity of the vessel (e.g. International Maritime Organization (IMO) number, Maritime Mobile Service Identity (MMSI) number, name).

In the literature, the term ‘vessel recognition’ is also found. Its precise meaning seems not to be the same for all authors, but it often overlaps with what is called here classification. The last step, identification, cannot generally be performed with the use of satellite imagery. Despite optimistic claims by some authors – e.g. Heiselberg (2016) proposes that a spectral library of ship signatures will enable vessel identification using Sentinel-2 multispectral images – we have seen no published proof of this, and it is hard to imagine that spectral or other signatures in optical satellite images can uniquely identify the hundreds of thousands of ships that exist in the world. The world merchant fleet alone counted over 87,000 ships in 2015 (EMSA, 2015), and only in a scenario where a-priori information leaves a few options, it may be possible that a satellite observation can decide on the identity of a ship. Therefore, vessel identification will not be comprehensively covered in this work, whereas the first two steps, detection and classification, as part of vessel detection workflow on optical images, will be taken up in detail (see Section 4).

### 1.1 Existing imaging systems for vessel detection

A large number of imaging systems can produce data for vessel detection. The most frequent image types can be divided into four categories: optical and reflected infrared, hyperspectral, thermal infrared, and radar. For optical and infrared images, two sub-categories can be further distinguished: video and night-time images; whereas for radar data, SAR (Synthetic Aperture Radar) can be additionally sub-categorised. These sensors can be deployed from various types of platform, and their usage is summarised in Table 1. The sensors may look downward, forward or sideways from their platform, and they have a wide range of complexity and operational cost.

**Table 1:**
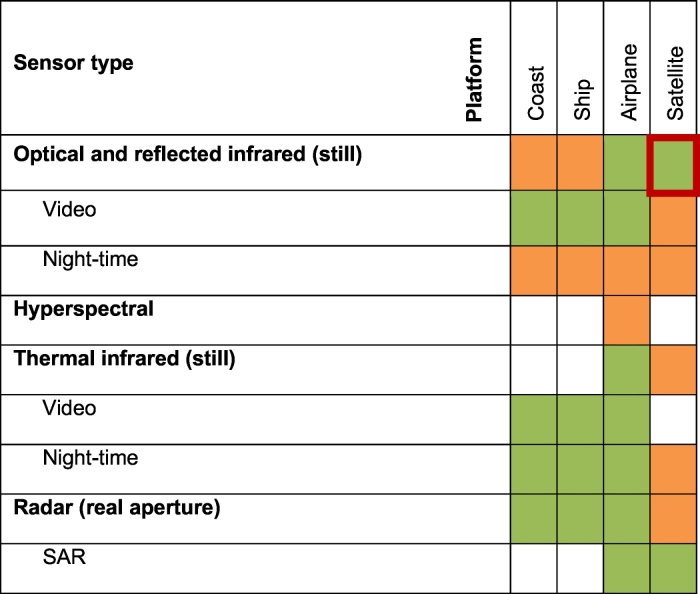
Combinations of imaging sensors and platforms that are used for vessel detection and maritime surveillance. A green field means the combination is very suitable and/or frequently used for vessel detection, whereas an orange field means it is only occasionally used for vessel detection. A white colour in the field means the sensor-platform combination is not generally used for detecting vessels. The red border marks the issue that is treated in this review.

**Fig. 1:**
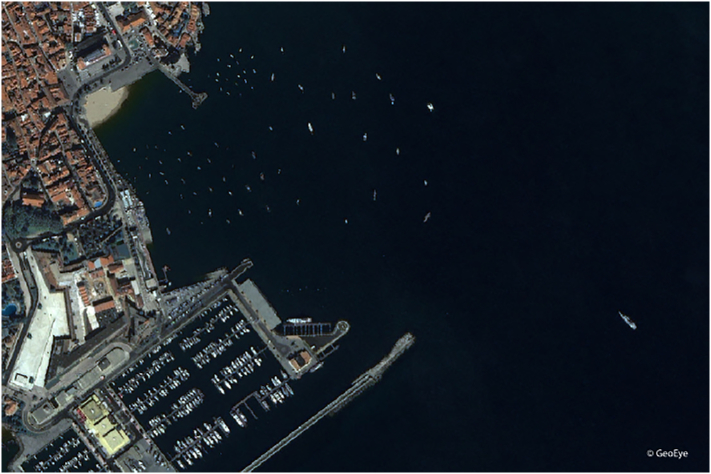
Detail of a GeoEye-1 optical image of Cascais (Portugal), acquired in October 2011, with a spatial resolution of 1.65 m (RGB composite). This image shows that vessels can be visually easily recognised from the image when there are no clouds or waves present, which implies that they can also be recognised by automatic processing methods.

Regarding the first sensor type in Table 1, optical and reflected infrared are grouped together because they have quite similar characteristics. Optical implies the visible spectrum detectable by the human eye (wavelengths approximately 400–700 nm), and reflected infrared covers the near- and short-wave infrared bands, up to 3 μm. These sensors are passive, meaning they rely on external illumination, generally from the Sun. The images are essentially photos (nowadays digital), and from a coastal location or from a ship they are partly being replaced by video (always in the context of vessel detection and maritime surveillance). However, for the satellite, this is still the main type of imaging sensor and the main topic of this review. Multi-spectral imagers can use the colour bands for more information, but they sacrifice on resolution which is higher for the panchromatic sensor. Optical images can provide valuable information for accurate vessel identification and feature extraction (Liu et al., 2013) and are relatively consistent. These images also enable classification and a wide application domain for their low price and simple structure (Lan and Wan, 2009). Optical Earth observation satellites with a resolution adequate for ship detection are usually located in low Earth orbit. A typical example of an optical satellite image of the coast is shown in Fig. 1. These systems observe the Earth top-down or slightly off-nadir (exceptionally further off-nadir). Their interpretation is relatively easy for a trained operator as the optical imaging technology works similarly to the human eye. They provide much information permitting vessel classification (Willhauck et al., 2005) because of their high spatial resolution and because of their spectral capacity, especially for vessels made of fiberglass, steel, aluminium, wood or concrete. When the targets are not extremely small (e.g. of mere few pixels), the spectral signature does play an important role in the ability to detect smaller targets (Pegler et al., 2007). Vessels have also a limited area, length and width range (they are commonly long and thin), which means that information valuable for their detectability and classification, besides their material, is also their size and shape. Nonetheless, because of several limitations of photo sensors (presence of high contrasts due to different weather conditions or sunlight effects, different angles of acquisition that can cause uncertain detection of the target, etc.), automatic vessel detection in photographs is still difficult, requiring more research in the area. Relevant factors of optical sensors are the following: revisit time, swath width, spatial, spectral and radiometric resolution, and incidence angle. Optical sensors perform acquisition at daytime, as long as there are good weather conditions (absence of heavy clouds). Bad weather conditions (clouds, winds and waves) and reflection of the sun on the water (sunglint) can strongly influence the detection performance. Regarding airborne platforms, recently there has been a growth of research into vessel detection from UAV (Unmanned Aerial Vehicle) (e.g. Dolgopolov et al., 2017, Xu et al., 2014).

Video cameras that detect mobile vessels are usually mounted on the coast, in ports, on aircraft/UAVs and sometimes on buoys or other floating platforms. Video-based techniques where sequences of image frames are analysed are well studied in road vehicle detection, but for vessel detection, video analysis still remains a challenging and complex task. Recently, several papers discussed the promising usage of video cameras to perform detection of vessels (Arshad et al., 2010, Bao et al., 2013, Fefilatyev et al., 2010, Hu et al., 2011, Rodriguez Sullivan and Shah, 2008). Camera-based vessel detection is attractive due to its low cost and ease of management, both in installation and maintenance. Optical video from satellite is just starting to emerge, but has great potential for ship detection because it can pinpoint moving targets.

With light-enhancement technology, video cameras are capable of providing imagery even in dark night conditions (Brown et al., 2005). Night-time optical sensors from coast or ship can be low-light detectors making use of ambient illumination, but from a satellite, this concerns mostly detection of shipborne lights. The spatial resolution of satellite imagers that are used for night-time observation is very low: 1 km at nadir for the Operational Linescan System (OLS) on board the Defense Meteorological Satellite Program (DMSP) satellites, and 375–750 m for the Visible Infrared Imaging Radiometer Suite (VIIRS) aboard the joint NASA/NOAA Suomi National Polar-orbiting Partnership (SNPP) satellite. Nonetheless, some publications have shown that in particular fishing ships can be detected by their nightlights from space; e.g. Cho et al. (1999) with OLS, and Elvidge et al. (2015), Lebona et al. (2016) and Straka et al. (2015) with VIIRS.

Hyperspectral sensors, in which an imaging spectrometer collects hundreds of narrow wavelength bands for the same spatial area, aim to exploit the spectral information content to the maximum. Such images are extremely complex and require advanced processing for analysis (González et al., 2013). It is a growing field in remote sensing, but they are not yet in general use. Their spatial resolution from orbit is too low to have allowed much development for ship detection, although a few attempts can be found (e.g. Marin-Mcgee, 2013, Wang et al., 2016).

Thermal infrared does not depend on solar illumination but on emission from the imaged objects themselves. It is therefore mainly attractive as night-time application. However, also during the day, temperature differences between ship (parts) and sea may allow detection (e.g. Lee et al., 1990, Leira et al., 2015, Mirghasemi et al., 2011, Neele, 2005, Wu et al., 2011). From aircraft, the moniker FLIR (Forward Looking InfraRed) is often used (also covering near-infrared), although such cameras may be pointed in any direction with a gimbal and they usually have a video function. From satellite, thermal infrared imaging is hampered by low resolution and by the atmosphere – concerning the latter not only by clouds but also by moisture.

Radar is a classic means for ship detection. It is most commonly exploited for navigation using rotating antennas on board of ships, and for vessel traffic control using antennas on the coast. Radar is an active sensor, meaning it is independent of external illumination. On an aircraft, a radar may use a scanning antenna or a fixed sideways looking antenna (SLAR; Side-Looking Airborne Radar). SLAR has also been used from satellite, but not often due to the low resolution, which also limits ship detection.

Synthetic Aperture Radar (SAR) is the type of radar that is suitable for use on satellites or airplanes, because its resolution is independent of the distance to the observed object. Where SLAR uses a long physical antenna to achieve spatial resolution, SAR uses the forward motion of the platform to synthesise a long antenna. SAR is probably the best available sensor for ship detection from satellite, because (a) achievable resolutions are matched to ship sizes (except for the very smallest ships), (b) it can image relatively wide areas at constant resolution, and (c) it functions independently of daylight or cloud cover. In addition, most (big) vessels are made of metal and their structure contains sharp edges that reflect radar signals intensively, so that the vessels on open waters come across as bright dots and edges (Máttyus, 2013). SAR satellite data have been readily available since the early 1990s, and by now there are a score of systems in orbit. Consequently, there is a vast body of research and literature on SAR ship detection (e.g. Crisp, 2004). Nonetheless, this method has a number of drawbacks: radar images are subject to a high level of intrinsic noise (speckle), ship detection is severely hampered by high wind and sea state, small targets are difficult to detect, false alarms are difficult to recognise, the classification of ship type is difficult and identification is impossible (Greidanus, 2005). However, the most important factor why ship detection from satellite SAR is not sufficient for adequate MDA is that the spatiotemporal coverage falls short by orders of magnitude. For adequate coverage, maybe hundreds of SAR satellites would need to be in operation instead of the dozen that exist now. There are, on the other hand, many more optical satellites in orbit. That is why vessel detection in optical satellite images can give an important contribution.

Although the technology behind airborne optical or infrared still or video cameras or SAR has different functionalities to those of optical satellite systems, there are some similarities in the processing techniques that can be utilised. However, in this article, we will focus only on optical imaging satellite systems. The review paper is organised as follows. Section 2 provides an overview and formation of literature that deals with optical vessel target detection and classification. In Section 3, we undertake the analysis of 119 papers, which cover both theoretical and practical points of view and allow us to obtain insights into the current state of the art. First, we show the correlation of existing optical Earth observation (EO) satellites and the number of papers on vessel detection through time. In Section 4, we thoroughly present every step that is important for a successful workflow, including sea-land separation, removal of environmental effects, vessel detection, discrimination and classification, and assessment of obtained results. We take into consideration the techniques associated with every step of processing optical satellite images. A special subsection is devoted to the fusion of optical and other available data in the maritime domain. In Section 5, we discuss and recognise some of the weak and strong points in today's research in vessel detection and classification, thus opening the possibility to predict future trends. Section 6 gives final conclusions.

## 2 Formation of literature database

This review paper analyses, compares and considers detection of vessels on high seas, in ports (inshore) and on inland waters from optical satellite sensors. Literature sources were searched for in Thomson Reuters Web of Science databases, ScienceDirect, and ResearchGate social media. The literature search was based on a predefined search of the following keywords: ‘ship detection’, ‘ship recognition’, ‘vessel detection’, and ‘vessel recognition’ in publication titles, keywords and abstracts (the search was performed in April 2017). References in all papers thus obtained were also examined and added to the overview, if missed before. The search period spanned over the last 39 years, starting from the first publication on vessel detection dated in 1978 (McDonnell and Lewis, 1978) and finishing in March 2017. We took into account only articles written in the English language.

Vessel detection is not only a topic in the domain of remote sensing but is quite interdisciplinary. We were able to find articles on the subjects of remote sensing, geo-informatics, signal processing, applied computer vision, robot vision, pattern recognition and artificial intelligence. However, only remote sensing technologies represent a source of large area satellite data while other domains provide useful concepts, methods, models, tools and insights for defining, visualising, querying and managing these data (Hay et al., 2003).

Finally, the 119 selected papers were analysed: 43 journal articles, 72 conference papers, 2 reports, 1 book chapter and 1 Master thesis. Until now, Zhu et al. (2010) is the most cited paper on vessel detection from optical satellite data in the SCI.

## 3 Inventory of evaluated studies

### 3.1 Structure of gathered literature

Table 2 gives an overview of the 119 selected publications sorted chronologically, based on the publication year. We have organised it according to ten key elements and issues, with the aim to provide the structure of the review. The meaning of columns is given below.

1. Year – the year in which the publication was published. In most cases, this means the research was done some time before, but the results were introduced to the public at a given time later. This usually correlates with the state-of-the-art satellite sensors of the given period.
2. Satellite sensor – type of optical satellite sensor used for the study, or a name of worldwide virtual mapping application where satellite data have been taken from (e.g. Google Earth, Microsoft Virtual Earth). In the latter case, authors usually do not give any significance to the particular satellite sensor or its metadata.
3. Band – defines which individual band or combination of bands was used to detect/discriminate/classify vessels. To acquire images of the Earth's surface, optical sensors use panchromatic mode (PAN) and/or multispectral mode (MS). In the latter, detection is done in several intervals of the visible, near-infrared (NIR) and short-wave infrared (SWIR) domain, with common bands in the visible being red (R), green (G) and blue (B). PAN images are recommendable for accurate geometrical measurements because of their higher spatial resolution, while the MS imagery is used for the vessel classification because of its spectral information. In cases where there is no notice about the bands used, we could reasonably infer the type of band (PAN or MS) from the resolution and/or sensor type mentioned in the text under consideration.
4. Image resolution – pixel size of an observed image expressed in meters, given for PAN and MS bands separately.
5. Min. size – minimal size of vessel that can still be detected with a given method (if mentioned). The detection of small vessels is of particular interest, since only larger vessels are regulated to be monitored by systems such as AIS and VMS (Johansson, 2011). Small vessel targets are generally represented only within a few pixels on a very high resolution (VHR) image. The interpretation of small vessels is not so straightforward, since it is sometimes difficult even for a trained interpreter to discern a vessel from other objects in the image. When there was an explicit mention of the fact that detection was oriented only towards big vessels (like cargo vessels, oil tankers, containers, etc.), we used the label ‘150 +’, meaning 150 m or more. Maximum vessel size can otherwise reach up to 400 m (the largest vessel currently in operation – the container ship Barzan – is measuring 400 m).
6. Methods of vessel candidate detection – descriptions of approaches to detect vessels from optical satellite images. These are only simple and short descriptions of usually complicated algorithms. These methods vary in their effectiveness and suitability depending on different images, scenes or resolutions. When no method is specifically mentioned, we used the label ‘n/a’.
7. Methods of discrimination/classification – descriptions of approaches to discriminate/classify vessels once they are detected (if used).
8. Eval. of results – states whether evaluation of the developed algorithm was carried out or not. Evaluation reflects the percentage of correctly detected (or classified) vessels and/or a false alarm rate.
9. Main purpose – the main purpose that has driven the development of the proposed algorithm or method. The label ‘Vessel detection’ is applied when either no particular purpose or a very general or diverse description for algorithm development was mentioned (e.g. detection of fishing, inshore, pirate, cargo, etc. vessels). Some papers deal with “inshore vessel detection” in ports and in inland waters (Beşbinar and Alatan, 2015, He et al., 2017, Hu et al., 2015, Li et al., 2016, Li et al., 2016, Liu et al., 2014, Ren et al., 2015, Xu et al., 2014), which, as opposed to vessel detection on open sea, has to cope with high similarity between vessels and port background in terms of colour and structure (Beşbinar and Alatan, 2015). The articles that developed algorithms not only for vessel detection but also for other geospatial object detection (such as airplanes, cars, storage tanks, baseball diamonds, bridges, etc.) fall under the label ‘Target detection’. Several authors (Han et al., 2014, Li and Itti, 2011, Lin et al., 2016, Wang et al., 2016, Yokoya and Iwasaki, 2015, Zhang et al., 2016) provide no explicit description of the vessel detection purpose but a general one for target detection. In the case of such occurrence, the analysis in this review takes into account only vessel detection.
10. Reference – list of main author(s). Full references are given in the References Section.

**Table 2:** Review of literature on vessel detection from optical satellite imagery (period 1978 – March 2017).

Year	Satellite sensor	Band	Image resolution [m] PAN MS		Min. size [m]	Methods of vessel candidate detection	Methods of discrimination/classification	Eval. of results	Main purpose	Reference
1978	Landsat-2 MSS	G, NIR	−	79	112	Threshold-based method: Threshold level definition	−	no	Vessel detection	McDonnell and Lewis
1993	SPOT XS, Landsat TM	G, NIR (SPOT), R, NIR (Landsat)	−	20 (SPOT), 30 (Landsat)	63 (SPOT), 108 (Landsat)	Transform-domain method: low and high pass filter, one-pass scan conversion algorithm	Discrimination: based on geometrical and spectral attributes	no	Tracking vessel movement	Burgess
2003	IKONOS	PAN, B, G, R, NIR	1	4	8	Shape and texture: local image statistics based on spatio-spectral considerations	Discrimination: length and width, spectral signature	no	Search and rescue	Pegler et al.
2005	SPOT-5	PAN	2.50	−	100	Shape and texture: shape constraints based method	−	no	Vessel detection	Wang et al.
2005	IKONOS, QuickBird	PAN	1 (IKONOS), 0.65 (QB)	−	10	Shape and texture: commercial software object-oriented methodology	Discrimination: based on length	no	Vessel detection	Willhauck et al.
2007	QuickBird	PAN	0.65	−	n/a	Threshold-based method: Histogram-based segmentation, Canny edge and Fourier transform	Discrimination: based on length and width	no	Vessel detection	Buck et al.
2007	Google Earth	n/a	7	−	n/a	Threshold-based method: Hierarchical cluster merging algorithm for multi-threshold image segmentation	Discrimination: based on geometrical and spectral attributes	no	Vessel detection	Hong et al.
2007	IKONOS	PAN, NIR	1	4	2	Shape and texture: spatio-spectral template enhanced with a weighted Euclidean distance metric	−	yes	Search and rescue	Pegler et al.
2008	QuickBird	PAN	0.65	−	n/a	Threshold-based method: Histogram-based segmentation, Canny edge and Fourier transform	Discrimination: based on length and width	no	Vessel detection	Buck et al.
2008	SPOT-5	PAN	5	−	14	Threshold-based method: adaptive threshold segmentation, region-growing segmentation	Classification: neural network	yes	Detection and classification	Corbane, Marre et al.
2008	SPOT-5	PAN	5	−	n/a	Threshold-based method: component tree image segmentation	Discrimination: Binary logistic regression	yes	Vessel detection	Corbane, Pecoul et al.
2008	n/a	n/a	n/a	−	n/a	Shape and texture: cumulative projection curve by using the Mahalanobis distance	−	no	Estimation of number of vessels	Hu and Wu
2009	Landsat-7 ETM +	PAN, B, G, R, NIR, SIR, MIR, Thermal	15	30	50	Threshold-based method: based on median spectral values	Discrimination: based on shape, texture and spectral characteristics	no	Vessel detection	Abileah
2009	QuickBird	n/a	−	2.50	n/a	Statistical method: graph partitioning active contours	Classification: Bayesian classifier	no	Vessel detection	Antelo et al.
2009	HJ-1	B	−	30	n/a	Transform domain method: Shannon theory for segmentation	Discrimination: based on spatial attributes	no	Vessel detection	Chen et al.
2009	VHR satellite images, sensor not mentioned	PAN	1		n/a	Shape and texture: region-based, shape-prior segmentation	Discrimination: similarity calculation	yes	Detection and classification	Tao et al.
2009	Landsat-5 TM	SWIR-1, SWIR-2	−	30	n/a	Statistical method: reflectance contrast calculation from PCA	−	no	Vessel detection, best band for vessel detection evaluation	Wu et al.
2010	unknown satellite images, Google Earth	n/a	n/a	n/a	n/a	Threshold-based method: Otsu segmentation, multi-resource extraction	Classification: SVM classifier based on shape and texture features	yes	Vessel detection	Bi et al.
2010	SPOT-5	PAN	5	−	20	Shape and texture: mathematical morphology	Discrimination: Binary logistic regression	yes	Vessel detection	Corbane et al.
2010	QuickBird	PAN	0.65	−	n/a	Shape and texture: grayscale morphological hit-or-miss transform with rank-order selection	−	no	Vessel detection	Harvey et al.
2010	Google Earth	B, G, R	n/a	n/a	150	Salient-based method: local gradient analysis, convexity of boundary and angle constraint	−	no	Vessel detection	Ma et al.
2010	SPOT-5	PAN	5	−	15	Statistical method: Bayesian decision theory	−	no	Vessel detection	Proia and Page
2010	CBERS, SPOT-2, SPOT-4, SPOT-5	G, R, NIR (CBERS), PAN (SPOT)	5, 10	20	n/a	Shape and texture: segmentation with global and local information, simple shape analysis	Classification: semi-supervised hierarchical classification, SVM	yes	Vessel detection	Zhu et al.
2011	n/a	n/a	−	−	n/a	Statistical method: graph-based fore/background segmentation, CFAR detector	−	no	Vessel detection	Chen et al.
2011	MODIS	bands 3–7	−	500	150 +	Statistical method: orthogonal subspace projection	−	no	Vessel detection	Dorado-Muñoz and Velez-Reyes
2011	SPOT-5, QuickBird, Google Earth,	PAN	10 (GE), 5 (SPOT-5), 0.6 (QB)	−	n/a	Shape and texture: splitting and merging segmentation method, texture roughness and ripple density of a MDC method	Discrimination: based on length to width ratio	yes	Vessel detection	Guang et al.
2011	SPOT-5	PAN	2.50	−	n/a	Shape and texture: based on statistical textures (LMP operator) and statistical histogram for sea-ship difference	Discrimination: based on confidence maps	yes	Vessel detection	Huang et al.
2011	Google Earth	B, G, R	−	0.5	< 10	Threshold-based method: component tree image algorithm	Classification: random forest classification	yes	Vessel detection	Johansson
2011	n/a (probably CBERS, SPOT)	n/a	10	10	n/a	Shape and texture: HSV colour space and local binary pattern method	Classification: SVM classifier	no	Vessel detection	Kumar and Selvi
2011	n/a	n/a	n/a	n/a	n/a	Salient-based method: saliency and gist feature extractor	Classification: SVM classifier	yes	Target detection	Li and Itti
2011	n/a	n/a	−	−	n/a	Computer vision method: scale-invariant feature descriptor with the visual Bag-of-Words method	−	yes	Vessel detection	Rainey and Stastny
2011	RapidEye	G	−	6.5	n/a	Threshold-based method: component tree technique	Discrimination: based on length to width ratio	no	Vessel detection	Saur et al.
2011	QuickBird, SPOT, IKONOS, Landsat	n/a	0.6–10	−	n/a	Threshold-based method: auto adapt multi-level threshold segmentation	Discrimination: based on geometrical attributes	yes	Vessel detection	Wang et al.
2011	GeoEye-1, aerial image	PAN, B, G, R	2.5	2.5	n/a	Shape and texture: Douglas optimisation, seed growing	Discrimination: seed growing	yes	Vessel detection	Wu et al.
2011	Google Earth	n/a	5 <	−	n/a	Shape and texture: Dynamic fusion model of multi-feature and variance feature	Classification: SVM classifier	yes	Vessel detection	Xia et al.
2011	Google Earth	B, G, R	0.6	−	n/a	Transform domain method: invariant generalised Hough transform using the evidence-gathering procedure	−	no	Vessel detection	Xu et al.
2011	Google Earth	n/a	n/a	−	n/a	n/a	−	no	Sea-land segmentation	You and Li
2011	n/a	n/a	n/a	−	n/a	Threshold-based method: Otsu segmentation, small minimum bounding rectangle shape analysis	−	yes	Vessel detection	Zuo and Kuang
2012	SPOT-5	PAN	5	−	50	Salient-based method: Neighbourhood similarity-based method	Classification: SVM classifier	yes	Vessel detection	Bi et al.
2012	Landsat-7 ETM +	B, G, R, NIR, SWIR-1, SWIR-2	30	−	n/a	Salient-based method: Phase spectrum of biquaternion Fourier transform	−	yes	Vessel detection	Ding et al.
2012	CBERS, SPOT	PAN	5 <	−	n/a	Transform-domain method: contrast box filter algorithm, scale invariant feature transform, K-means algorithm	Classification: SVM classifier	yes	Vessel detection	Guo and Zhu
2012	n/a	n/a	n/a	−	150 +	Threshold-based method: line segment detection with region growing algorithm	Discrimination: different gradient orientation	yes	Vessel detection	Lin et al.
2012	CBERS, SPOT	PAN, B, G, R	10	10	n/a	n/a	Classification: SVM classifier	no	Vessel detection	Satyanarayana and Aparna
2012	QuickBird, OrbView	n/a	0.65 (QB), 1 (OV)	4	n/a	Threshold-based method: component tree image segmentation	Classification: Fisher classifier	yes	Vessel detection	Zhang et al.
2013	Google Earth	n/a	< 4	−	150 +	Shape and texture: multi-feature calculation	Discrimination: Dempster-Shafer evidence theory	no	Vessel detection	An et al.
2013	GeoEye-1	PAN, B, G, R, NIR	0.5	2	n/a	Computer vision method: least median squares filter, scale-normalised Laplacian and watershed segmentation	−	no	Vessel detection	Bouma et al.
2013	WorldView-2	Coastal, B, G, Yellow, R, RedEdge, NIR1, NIR2	0.5	1.6	n/a	Anomaly detection method: continuum fusion derived anomaly detector	−	no	Vessel detection	Daniel et al.
2013	GeoEye-1	n/a	0.5	2	n/a	Statistical method: constant false alarm rate detector	Discrimination: based on geometric, statistical and spectral characteristics	no	Vessel detection	Dekker et al.
2013	Google Earth	n/a	n/a	−	n/a	Salient-based method: optical flow and saliency method	Discrimination: based on area	no	Vessel detection	Deng et al.
2013	Google Earth	n/a	n/a	−	n/a	Threshold-based method: Otsu segmentation, shape and texture: Chamfer matching algorithm	Discrimination: shape analysis	yes	Vessel detection	Dong et al.
2013	WorldView-1, − 2	n/a	0.5	−	8	Computer vision method: pattern recognition methods	−	no	Vessel detection	Máttyus
2014	QuickBird, Google Earth	n/a	n/a	−	n/a	Salient-based method: visual saliency detection, Chan-Vese segmentation model	Classification: dynamic probability generative classification model	yes	Vessel detection	Guo et al.
2014	Google Earth	B,G,R	0.5–2	−	n/a	Salient-based method: training of the visual attention, Fisher discrimination dictionary learning	−	yes	Target detection	Han et al.
2014	n/a	PAN	0.5	−	n/a	Salient-based method: Harris corner detector and Local salient region analysis	−	no	Vessel detection	Jin et al.
2014	SPOT-4, SPOT-5, FORMOSAT2, KOMPSAT2, Pleiades	PAN	0.5–150	−	n/a	Transform domain method: discrete wavelet transform, constant false alarm rate detector	Discrimination: multiscale discrimination algorithm	yes	Vessel detection	Jubelin and Khenchaf
2014	GeoEye-1, WorldView-2, IKONOS, QuickBird	B, G, R, NIR	0.4 (GE), 0.5 (WV), 1 (IK), 0.6 (QB)	1.6 (GE), 1.8 (WV), 4 (IK), 2.5 (QB)	4	Threshold-based method: histogram-based segmentation, simple statistics	Discrimination: based on spectral characteristics	yes	Vessel detection	Kanjir et al.
2014	QuickBird	PAN	0.65	−	n/a	Shape and texture: Set of shape analysis	Discrimination: based on geometric and context information	yes	Vessel detection	Liu et al.
2014	ASTER VNIR	G, R, NIR	15	−	15	Shape and texture: Quad tree decomposition, extraction through bounding rectangular patches	Discrimination: based on size and elongation	no	Vessel detection	Partsinevelos and Miliaresis
2014	Google Earth	PAN	1	−	40	Anomaly detection method: hyperspectral anomaly detector Reed-Xiaoli	Classification: Adaboost classifier	yes	Vessel detection	Shi et al.
2014	n/a	n/a	n/a	−	n/a	Salient-based method: Itti-Koch visual attention model, local binary pattern feature extractor	Classification: SVM classifier	yes	Vessel detection	Song et al.
2014	Google Earth	n/a	0.5, 1	−	n/a	Threshold-based method: based on textural and geometric features	Classification: SVM classifier	yes	Vessel detection	Xia et al.
2014	Google Earth	n/a	0.6	−	n/a	Transform domain method: robust invariant generalised Hough transform	−	yes	Vessel detection	J. Xu et al.
2014	SPOT-5, Google Earth	PAN	5	−	n/a	Anomaly detection method: linear function combining pixel and region characteristics	Discrimination: based on geometric characteristics	yes	Vessel detection	Yang et al.
2015	IKONOS, GeoEye, QuickBird, Worldview	PAN	0.5, 1	−	n/a	Shape and texture: local binary patterns	Classification: decision-level classifier using majority voting algorithm	yes	Vessel detection	Fernandez Arguedas
2015	n/a	PAN, B, G, R, NIR	0.5	0.5, 4	n/a	Threshold-based algorithm: line segment detector with group-and-merge method	Discrimination: based on shape, SVM classifier with RBF kernel	no	Inshore vessel detection	Beşbinar and Alatan
2015	Google Earth	n/a	−	−	n/a	Shape and texture: histogram of oriented gradients based on rotating detection window	Discrimination: SVM classifier	yes	Vessel detection	Gan et al.
2015	n/a	n/a	−	−	n/a	Computer vision method: co-training model	−	yes	Vessel detection	Guo, Wang, Xia
2015	Quickbird, Google Earth	n/a	−	−	n/a	Transform domain method: improved Hough transformation, rough set theory	Classification: dynamic probability generative model	yes	Vessel detection	Guo, Xia, Wang (a)
2015	Google Earth	n/a	−	4	n/a	Statistical method: entropy-based hierarchical discriminant regression	−	yes	Battlefield vessel detection	Guo, Xia, Wang (b)
2015	n/a	n/a	2	−	n/a	Transform domain method: Hough Transform	−	no	Inshore vessel detection	Hu et al.
2015	n/a	n/a	−	−	n/a	Computer vision method: mutual information random forest method	−	yes	Vessel detection	Huang et al.
2015	Geoeye-1	PAN	−	−	n/a	Shape and texture: region growing method on binary image, morphological operators	−	no	Vessel detection	Jin and Zhang
2015	Google Earth	n/a	−	3	n/a	Shape and texture: histogram of oriented gradients	Discrimination: adaptive target filter	yes	Vessel detection	Ju
2015	Google Earth	n/a	−	−	n/a	Salient-based method: multi-layer sparse coding model, Lasso and Otsu method, deformable part model	Discrimination: SVM classifier	yes	Vessel detection	Li et al.
2015	Google Earth	PAN	0.61	−	n/a	Shape and texture: composite Kernel support Vector machine	Classification: SVM classifier	yes	Vessel detection	Pan et al.
2015	Gaofen-1	PAN	2	−	n/a	Transform domain: phase spectrum of Fourier transform and adaptive segmentation	Classification: histogram of oriented gradients	yes	Vessel detection	Qi et al.
2015	Google Earth	n/a	−	0.6	n/a	Salient-based method: the basic Harris and SUSAN method	−	yes	Inshore vessel detection	Ren et al.
2015	Google Earth	B, G, R	−	−	n/a	Salient-based method: Itti model, local texture histograms extraction by Local Binary Pattern, Gabor filters	Discrimination: SVM classifier with RBP kernel	yes	Vessel detection	Shi et al.
2015	CBERS, SPOT4	n/a	5, 10	20	n/a	Shape and texture: histogram of gradient and pyramid binary pattern feature	Classification: SVM classifier based on RF fingerprints	yes	Large scale vessel detection	Sun et al.
2015	SPOT-5	PAN	5	−	50	Deep learning method: compressed-domain framework, deep neural network and extreme learning machine	Classification: deep neural network	yes	Vessel detection	Tang et al.
2015	WorldView-2, QuickBird-2, GeoEye-1, RapidEye, Formosat-2	PAN, B, G, R, NIR	0.5 (WV), 0.6 (QB), 0.4 (GE), 2 (FS)	1.8 (WV), 2.5 (QB), 1.6 (GE), 6.5 (RE), 8 (FS)	n/a	Shape and texture: Minimum Noise Fraction algorithm, object-based image analysis	Discrimination: threshold rule set classification	no	Vessel detection for search and rescue operations	Topputo et al.
2015	n/a	n/a	−	−	n/a	Salient-based method: based on linear iterative clustering and cellular automata, maximum contrast of image patch	−	no	Vessel detection from MS and SAR data	Wang et al.
2015	Landsat 5 TM, Landsat 7 ETM +	SWIR 2	−	30	n/a	Threshold-based method: threshold segmentation	−	no	Vessel and oil platform detection	Xing et al.
2015	SPOT5	PAN	5	−	n/a	Salient-based method: global contrast model	Discrimination: based on shape, texture and neighbourhood similarity, LPB-SVM classifier	yes	Vessel detection	Yang et al.
2015	WorldView-2	PAN	0.5	−	n/a	Computer vision method: sparse representation and Hough voting (SR Hough)	−	yes	Target detection	Yokoya and Iwasaki
2016	n/a	n/a	−	−	n/a	Deep learning method: artificial neural network	−	no	Vessel detection comparing GPRS and satellite images	Amabdiyil et al.
2016	Sentinel-2	B, G, R, NIR	−	10	n/a	Shape and texture: commercial software object-oriented methodology	−	no	Vessel detection on Danube river	Dana Negula et al.
2016	WorldView-2, QuickBird-2, GeoEye-1	n/a	−	10 (under-sampled)	20.0	Statistical method: object-based image analysis - Gaussian modelling of CFAR	−	yes	Vessel detection from MS and SAR data	Gianinetto et al.
2016	n/a	n/a	−	−	n/a	Salient-based method: Itty-Koch with gradient, combined visual local binary pattern	Discrimination: based on textural statistical features	yes	Vessel detection	Haigang and Zhina
2016	Sentinel-2	B, G, R, NIR	−	10	30.0	Threshold-based method: minimum threshold between the background and ship reflectances	Discrimination: based on geometrical and spectral characteristics	no	Vessel recognition and velocity determination	Heiselberg
2016	VNREDSat-1	PAN	2.5	−	n/a	Threshold-based method: based on abnormality score of objects	Discrimination: based on shape, texture and spectral characteristics, PCA	yes	Vessel detection	Hung
2016	Google Earth	n/a	−	−	n/a	Shape and texture: mathematical morphology and graph partitioning active contours segmentation	Classification: active deep network based on best versus second-best method	yes	Vessel detection	Huang et al.
2016	n/a	n/a	15	−	n/a	Statistical method: Gaussian and median filter, adaptive iterative segmentation	Discrimination: based on geometric characteristics	yes	Real-time on-board vessel detection	Ji-yang, Dan, Lu-yuan, Jian, Yan-hua
2016	n/a	n/a	−	−	n/a	Salient-based method: multi-scale enhancement method (wavelet decomposition and Otsu method)	−	yes	Real-time on-board vessel detection	Ji-yang, Dan, Lu-yuan, Xin, Wen-juan
2016	SkySat-1	n/a	1.1 (video)	−	n/a	Threshold-based method: Otsu segmentation, Gabor features, intensity, Fourier transform	−	no	Vessel detection from video on optical satellite	Li and Man
2016	WorldView-1	PAN	0.5	−	25.0	Shape and texture: shape parameters	−	yes	Vessel detection	N. Li et al.
2016	Google Earth	n/a	−	1	n/a	Transform domain method: transform domain and SVM classifier, saliency of directional gradient information	Discrimination: based on context information	yes	Inshore vessel detection	S. Li et al.
2016	Google Earth	n/a	−	−	n/a	Salient-based method: multi-scale fractal dimension feature by using differential box counting method	−	yes	Vessel detection in a large scene	W. Li et al.
2016	CBERS-02B	PAN	2.36 (HR camera)	−	n/a	Statistical method: Based on surface fitting method (Gaussian distribution model)	Discrimination: morphological filtering	yes	Vessel detection in a large scene	X. Li et al.
2016	Google Earth	n/a	−	1	n/a	Computer vision method: rotation and scale invariant method based on the pose-consistency voting	−	yes	Target detection	Lin et al.
2016	Gaofen-1	PAN, B, G, R	2	8	n/a	Shape and texture: commercial software object-oriented methodology	Discrimination: based on length and width	no	Inshore vessel detection	B. Liu et al.
2016	Landsat-8	IR, TIR	15	30	n/a	Transform domain method: Discrete wavelet transform	Discrimination: morphological filtering	yes	Vessel detection	Y. Liu et al.
2016	Google Earth	Google Earth	−	−	n/a	Statistical method: rotated bounding box space	Discrimination: binary linear modelling	yes	Vessel detection	Z. Liu et al.
2016	Google Earth	n/a	−	−	n/a	n/a	Discrimination: attribute-based model	yes	Vessel category recognition	Oliveau and Sahbi
2016	Google Earth, Gaofen-1	n/a	−	1 (GE), (GF-1)	n/a	Threshold-based method: Otsu segmentation, morphological operations	Discrimination: based on geometric characteristics	yes	Vessel detection	Shuai, Sun, Shi, Chen
2016	Google Earth	n/a	−	−	n/a	Computer vision method: scale invariant feature algorithm	Discrimination: maximum match number with library	yes	Vessel detection	Shuai, Sun, Wu et al.
2016	WorldView-2, QuickBird-2, GeoEye-1, RapidEye, Formosat-2, Sentinel-2	PAN, B, G, R, NIR	0.5 (WV), 0.6 (QB), 0.4 (GE), 6.5 (RE), 2 (FS)	2 (WV), 2.4 (QB), 1.6 (GE), 5 (RE), 8 (FS), 10 (S2)	0–15	Shape and texture: Minimum Noise Fraction algorithm, object-based image analysis	Classification: threshold-rule set classification	yes	Vessel detection for immigrant search and rescue	Topputo et al.
2016	Google Earth	n/a	−	−	n/a	Computer vision method: scale invariant feature transform descriptor with improved bag-of-words model	Discrimination: phase spectrum of quaternion Fourier transform	yes	Target detection	X. Wang et al.
2016	Orbview-3	PAN, B, G, R	1	4	n/a	Anomaly detection method: Probability density function, vessel distribution by the density	Discrimination: structural continuity descriptor (based on width to length ratio)	yes	Vessel detection on open sea	Xiaoyang et al.
2016	Google Earth	PAN	−	−	n/a	Salient-based method: hypercomplex frequency domain and phase quaternion Fourier transform	Discrimination: radon transform and histogram of oriented gradients	yes	Vessel detection	Xu and Liu
2016	n/a	PAN	2	−	40.0	Computer vision method: AdaBoost classifier trained by Haar features, Line Segment Detector	−	yes	Vessel detection	Yao et al.
2016	Microsoft Virtual Earth	n/a	−	4	n/a	Salient-based method: phase spectrum of Fourier transform saliency and frequency-tuned saliency	Classification: based on geometric characteristics, SVM classifier	yes	Vessel detection	Yin et al.
2016	Google Earth	n/a	0.5, 1	−	n/a	Salient-based method: normal directional lifting wavelet transform	−	yes	Target detection	L. Zhang et al.
2016	Google Earth	n/a	0.12, 0.25	−	n/a	Deep learning method: ship proposal extraction convolution neural networks	−	yes	Vessel detection	R. Zhang et al.
2016	GaoFen-1, VRRS-1, Google Earth	PAN	2 (GF-1), 16 (VRSS-1)	−	20 pixels	Deep learning method: convolutional neural network, Singular value decomposition algorithm	Discrimination: SVM classifier	yes	Vessel detection	Zou and Shi
2017	Google Earth	n/a	−	−	n/a	Computer vision method: rotation and scale-invariant method based on the pose consistency voting	−	yes	Inshore vessel detection	He et al.
2017	Google Earth	PAN	2	−	n/a	Salient-based method: maximum symmetric surround method, cellular automata dynamic evolution model, Otsu algorithm	Discrimination: histogram of oriented gradient, AdaBoost classifier	yes	Vessel detection	Wang et al.
2017	Google Earth	B, G, R	−	−	10 pixels	Salient-based method: combined saliency map model through a self-adaptive threshold based on Entropy information	Discrimination: based on gradient features	yes	Vessel detection	Xu et al.
2017	Google Earth	PAN	2	−	n/a	Salient-based method: Histogram-based contrast method, phase spectrum of a Fourier transform, surface regular index	Discrimination: Simple shape analysis, structure-local binary pattern, AdaBoost algorithm	yes	Vessel detection	Yang et al.

Table 2 aims to present objective information, but inevitably the summary is influenced by the authors' personal views. Some papers may not discuss the above-mentioned categories directly, in which case the categorisation for Table 2 represents the subjective choice of the authors of this study. We included any forms of vessel detection into our review since we wish to represent a variety of approaches and solutions for the vessel detection methodologies and methods, and examine how they developed over the last decade. A few studies have also dealt with vessel wake and track detection from optical satellite images (Campmany et al., 2009, Rao et al., 2005); however, if they did not include vessels as prime detection, they have not been included in the review.

### 3.2 Relation of publications and optical satellites

**Fig. 2:**
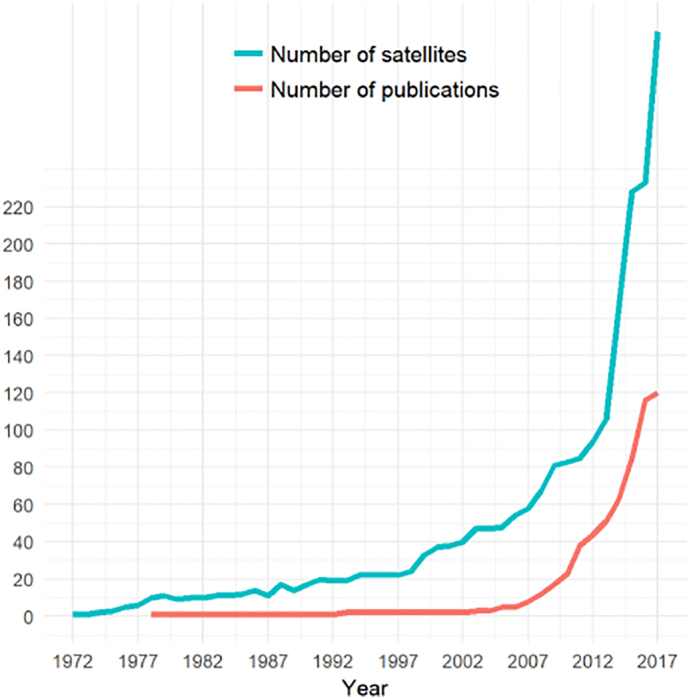
The number of publications on vessel detection (lower curve) has been rising following the increase of available optical satellites in orbit (upper curve). The number of publications inserted to represents all of the published papers on vessel detection in English which have been found and analysed by the authors of this research. The year 1972 marks the launch of the first civil optical satellite Landsat 1. The data on satellite launches are adapted and updated from Belward and Skøien (2015).

In this section, we describe existing optical satellite sensors used for vessel detection in relation to the number of publications across time. The first civil optical satellite was launched in 1972 (Landsat 1) and until today, hundreds of optical satellites with a broad range of resolutions have been orbiting our planet. Although much more work on automatic vessel detection has been performed on SAR data up to now, the first research of vessel detection from optical imagery was already done in 1978 (McDonnell and Lewis, 1978); the same year as the vessel detection on SAR data was first demonstrated by the experimental SEASAT. The detection from both types of images therefore started more or less at the same time. Radar satellite imagery started to be used extensively in automatic detection of vessels since the launch of the European Remote Sensing satellite ERS-1 in 1991, whereas optical data became popular only a decade later when VHR satellite systems became widely commercially available. In many cases, these spacecraft do not undertake image acquisitions over oceans, since covering all of the Earth's surface would significantly increase the volume of data to be transmitted to the ground. Also, revenue for commercial systems is usually earned by generating imagery of land (Bannister and Neyland, 2015) or coast. Fig. 2 shows the relation of the number of optical satellites in existence and the number of existing publications on vessel detection on optical satellite images across time. Both segments are seen to increase ever more strongly from the year of the first optical satellite launch until March 2017 (the end of this study).

A noticeable change happened in the new millennium, when commercial VHR optical satellites started being widely available, offering cutting-edge spatial resolution by reaching a Ground Sample Distance (GSD) lower than 5 m at nadir. Consequently, the number of publications increased drastically. Before that period, only a few optical satellites, offering limited passes, were available, and success was thus restricted to the rare chances when satellite overpass coincided with clear skies (Fingas and Brown, 2011). With such a prerequisite, the probability of detecting a vessel on a satellite image was low. During the last 17 years, and especially in the last couple of years due to numerous launches of micro- and nano-satellites, the number of optical VHR satellite missions has been growing, improving the temporal resolution and enabling frequent monitoring of the same areas. However, it should be noted that because of the narrow swath (spatial coverage) and relatively high costs in the past, VHR images were generally utilised in small regions, and were hardly applied to larger areas. While VHR is required to detect a vessel, large fields of view are required to maximise the probability of a vessel being captured at a given time and location (Bannister and Neyland, 2015).

**Fig. 3:**
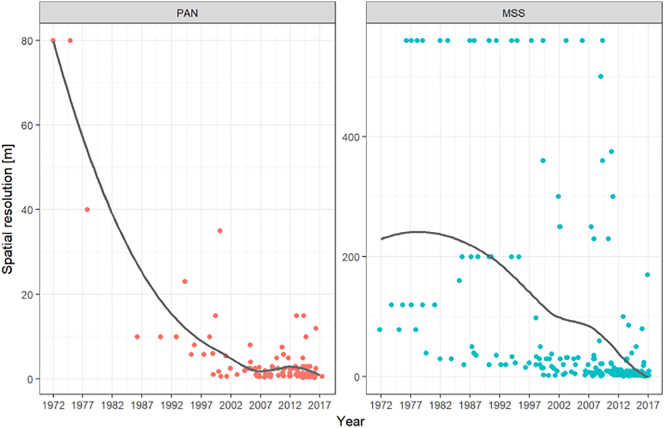
The increase of spatial resolution of panchromatic and multispectral sensors through the years. Dots represent an existing satellite and its highest available resolution, and the line represents the moving average. The higher the resolution (i.e. the smaller the size of the pixel), the more information can be extracted from the image.

Recent VHR satellites such as the WorldView and GeoEye series and Pleiades not only provide a more frequent coverage of the Earth than the previous satellites, but also higher spatial and spectral resolutions. Fig. 3 shows the increasing spatial resolution (or decreasing pixel dimensions) of optical satellite sensors through time for both PAN and MS sensors. Higher spatial resolution, on the one hand, allows detection of greater details in the image (smaller vessels, and diverse features on big vessels) enabling a more complex analysis, and on the other presents a bigger processing challenge due to the larger quantity of data.

Apart from the increase in temporal and spatial resolution, the radiometric resolution, expressed in number of bits, has also improved. We were unable to find any research proving that higher radiometric resolution results in better vessel detection, although the general knowledge says that higher radiometric resolution enables differentiation of smaller details in the images, meaning in our case better detection of vessels on the sea. Both the background (all non-vessel pixels) and the vessels can exhibit highly diverse intensities in the image, therefore a bigger bit-depth can be beneficial. In practice, the intensity of the same vessel may change from image to image and may even appear darker than the background, and the background can take different appearances, depending on the wind, the swell and the capturing conditions.

Revisit times of satellites have also substantially decreased. Some of the satellites are able to point their sensors to the same area between different satellite passes (e.g. WorldView-2), others are launched in constellations in order to be able to increase revisit frequency (e.g. RapidEye, Sentinel). For example, with two optical Sentinel-2 satellites in orbit it is possible to observe coasts every 5 days at the Equator and every 2–3 days at mid-latitudes (Aschbacher and Milagro-Pérez, 2012) – which is however still surpassed by the pair of Sentinel-1 radar satellites that reach 1–3 days thanks to their wider image swath and day/night operation.

## 4 Analysis

### 4.1 Sensors used

The most frequently used optical sensors in the literature on vessel detection from the VHR class (up to 5 m resolution, and many have sub-meter resolution) are QuickBird, IKONOS, WorldView-2, GeoEye-1 and recently WorldView-3; from the HR class (from 5 to 20 m spatial resolution) it is SPOT-5; while from medium resolution (where pixels are bigger than 20 m) the most used are Landsat and CBERS. In the last years studies using Gaofen-1, Formosat-2 and Sentinel-2 sensors increased. Less frequently tested sensors in the literature on vessel detection are HJ-1A, MODIS, OrbView-1, Aster VNIR, VRRS-1, Kompsat-2, SkySat-1 and Pleiades.

Night-time visible and IR images provide an alternative and complement especially for fishing vessel detection. Some research for vessel detection has been conducted using the Visible Infrared Imaging Radiometer Suite (VIIRS) aboard the joint NASA/NOAA Suomi National Polar-orbiting Partnership (SNPP) satellite (e.g. Elvidge et al., 2015, Lebona et al., 2016, Straka et al., 2015) and the Operational Linescan System (OLS) on board the DMSP (Defense Meteorological Satellite Program) (e.g. Cho et al., 1999). VIIRS, which collects data both by daytime and night-time each day across the globe, has a spectral band that spans from 0.5 to 0.9 μm, straddling the visible and near-infrared (Elvidge et al., 2015). At night, it is especially useful to detect fishing boats that use lights to attract catch almost in near-real time. In 2014, NOAA's Earth Observation Group (EOG) began developing algorithms to report, with low temporal latency, the locations of boats detected based on lights from VIIRS globally (NOAA, 2017). One drawback for use of night-time visible or IR images is their low spatial resolution; VIIRS has a spatial resolution of 750 m at nadir, and OLS has 1 km, so they can detect only brightly lit vessels, and several boats could be present in a single pixel. Additionally, it is not possible to distinguish different types of lighting, for example, LED vs. incandescent (Elvidge et al., 2015), which could help to assess vessel classes.

A relatively large fraction of the authors (35%) used Google Earth images as the direct input data source in their research on vessel detection – either as exported data or simply as a print screen –, or as a source for collecting the greater amount of test data for machine learning methods (An et al., 2013, p. 201; Deng et al., 2013, Dong et al., 2013, Gan et al., 2015, Guo et al., 2015, Han et al., 2014, Hong et al., 2007, Huang et al., 2016, Johansson, 2011, Ju, 2015, Ma et al., 2010, p. 201; Shi et al., 2014, Xu et al., 2017, Xu et al., 2011, p. 201; Xu and Liu, 2016, Xu et al., 2014, Yang et al., 2017, Yang et al., 2014, You and Li, 2011, Zhang et al., 2016, Zou and Shi, 2016). One author has used data from Microsoft Virtual Earth (Yin et al., 2016). The use of mentioned data is especially increasing in last years. Google Earth/Microsoft Virtual Earth images are freely available for anyone to view from the application imagery and for personal use. They offer spectral, texture, geometry and other characteristics for analysis, and, as stated by Liu et al. (2017), experiments on images derived from Google Earth are important references to practical applications with optical data directly collected from spaceborne devices. But since these images are pre-processed for visualisation purposes only, they have relatively poor spectral information (with red, green and blue bands), and also large inconsistent radiometric distortions (Yu and Gong, 2012). In addition, positional accuracies are not very good and not always consistent, indicating that these data are questionable for any profound remote sensing analysis. Images from worldwide virtual mapping applications should therefore be treated as ancillary data to collect the training or testing samples for vessel detection and classification, or to be used for validation or visualisation of detected vessels (if the image is the same). Google Earth/Microsoft Virtual Earth images can also serve as a source for image search.

Contrary to those images, each of the aforementioned optical sensors produces MS imagery that is composed of several bands, usually red, green, blue and NIR, sometimes even more, and most of these sensors also include a PAN band. For vessel detection, different combinations of spectral bands can be used.

### 4.2 Vessel detection workflow on optical images

**Fig. 4:**
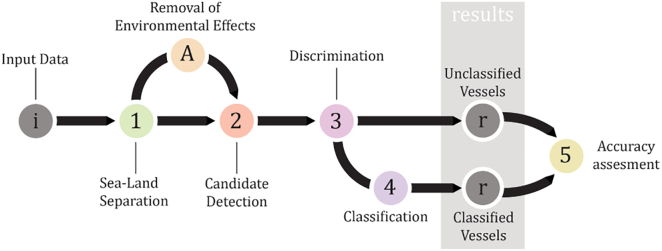
A common scheme of vessel detection workflow (generalisation from all gathered literature).

The vessel detection problem on optical images can be simply considered as a rather straightforward detection of bright points against a darker background. The reality is however much more complicated; it may happen that a ship is darker than the surrounding sea surface, and there may be many other bright objects present in the image that can be wrongly detected as vessels. A large part of the gathered literature on vessel detection divides their processing workflow into few basic steps. In the main workflow (Fig. 4) the first step is the sea-land separation where land areas are removed from the image, and thus neglected in further processing. (In some cases of inshore detection, where the moored ships are connected to the land, the first step is instead the removal of the sea pixels by thresholding, while the main algorithm later separates the ship targets from the land pixels). Only a few authors applied the next step, the removal or mitigation of environmental effects (e.g. sunglint, clouds, waves), in the algorithm. Following that, the most important part of the workflow is vessel candidate detection, in which the vessel candidate objects are obtained. Candidates are then discriminated from the rest of the detected, non-vessel objects. Discrimination may be upgraded through classification where vessels are further assigned into more detailed vessel classes according to certain attributes. Acquired results are then assessed, thus making up for the last section in the vessel detection workflow. Explanations for all the main sections are given in detail below.

#### 4.2.1 Sea-land separation

An accurate sea-land separation (called also land masking) is a relevant step in the vessel detection. It is not only necessary for an accurate detection of vessels in harbour areas (Willhauck et al., 2005), but is also crucial because vessel detectors can produce high numbers of false alarms when applied to land (Corbane, Pecoul et al., 2008). Sea-land separation falls into two groups: by importing an external mask into the system or by creating it from the image itself. Around 40% of all the authors have applied and described their sea-land separation procedure.

Coastline data, describing the line that separates a land surface from an ocean or sea (Lavalle et al., 2011) can be imported as existing GIS data. The most used dataset is the freely available world shoreline database, which is a combination of the world data bank (WDB) and the world vector shorelines (WVS) databases. They are merged in the Global Self-consistent, Hierarchical, High-resolution Shoreline (GSHHS) database (more recently renamed as GSHHG – Global Self-consistent, Hierarchical, High-resolution Geography) that is available for free on the Internet (NOAA, 2017). Many algorithms for vessel detection use this shoreline shapefile for land masking at a scale 1:250.000 as an overlay onto an image (Buck et al., 2008, Buck et al., 2007, Corbane et al., 2008, Corbane et al., 2008, Daniel et al., 2013, Zou and Shi, 2016), whereas Jin and Zhang (2015) have used instead NOAA's medium resolution coastline at a scale 1:70.000 for higher details. Another freely available global water mask is the one that uses the Shuttle Radar Topography Mission (SRTM) and the SRTM Water Body Data (SWBD) dataset for coastlines between ± 60° latitude with a resolution of around 30 m. This one was used as a binary land mask by Máttyus (2013).

For image-based land masking, many authors used simple histogram-based segmentation (Bi et al., 2010, Hung, 2016, Kanjir et al., 2014, Li et al., 2016, Lin et al., 2012, Pegler et al., 2007, Tang et al., 2015, Wang et al., 2005, Wu et al., 2011, Xu et al., 2014, Xu et al., 2011, You and Li, 2011), also known as histogram threshold or Otsu method. Water has a low reflection in the image histogram and can therefore be relatively easily removed. The image is then segmented into water and non-water according to the threshold. This method is simple and fast, especially if VHR images are first downsampled to reduce the computational load. The procedure, however, can produce some errors, e.g. leave areas of land out of the mask or remove vessels with low intensities from the sea. These can be corrected to some extent by using filtering and/or by smoothing of images; morphological filters are typically used to connect isolated segments and delete isolated pixels. The accuracy of the segmentation determines the accuracy of vessel detection. To improve the results of the sea-land mask using histogram-based segmentation, Beşbinar and Alatan (2015) have additionally used Digital Terrain Elevation Data (DTED) to generate a precise sea-land mask by utilising zero values.

Burgess (1993) used a heuristic approach for land masking based on observations of the relationship between the data values in the two input images for both class types (sea, land). She also applied a low-pass and a high-pass filter on the masked images to obtain an improved land mask. Tang et al. (2015) first separate the image into segments of two types, (1) land + ship and (2) sea, and then use size and shape to distinguish ship segments from land segments. Zhu et al. (2010) included image segmentation with global and local information (the characteristics of grey and edge features), not only to mask land areas from the image but also to mask everything that is not a vessel candidate. The two-part algorithm for sea-land segmentation was implemented by Xia et al. (2011). This multi-feature fusion model in the first part extracts features from the source image using texture and grey features to get two different feature maps. In the second part, authors fuse these maps into an integrated normalised feature map. Combining them allows calculation of the dynamic feature weight matrix. The result of the procedure is a grey image on which a certain threshold is set in order to obtain background (land) and sea area. Dong et al. (2013) implemented a land mask based on the texture difference between the sea and the land. Combining different texture features (contrast, smoothness, entropy, …) they applied a region growing method to segment both classes. Another method for land masking was developed by Liu et al. (2014), using shape and texture information. They proposed an energy function based on an active contour model, which is an improved threshold Otsu method. You and Li (2011) concentrated on improving the threshold between land and sea using an adaptively established statistical model of the sea area. Land that is incorrectly classified is removed according to the difference of variance in the statistical model. From all of the accessible publications, only very few (Johansson, 2011, Topputo et al., 2016) manually removed areas of land from the image.

#### 4.2.2 Removal or mitigation of environmental effects

The presence of environmental factors in optical images is an undesirable, but generally unavoidable fact. The main environmental factors that significantly influence vessel detection accuracy encompass waves, cloud coverage and sunlight reflection on the water (sunglint). Environmental effects – especially if there are many – may complicate vessel detection to various degrees and thus may strongly influence the detection accuracy. For example, Yang et al. (2014) tested their performance on the different sea surfaces (quiet, textured and cluttered seas) and they have shown that accuracy decreases drastically from a quiet sea to a cluttered sea. Only few authors included the removal of environmental effects separately into the algorithm. As these effects can be diminished with the use of an appropriate vessel candidate detection methodology itself, this step is shown as a separate one and as optional in Fig. 4. A few examples are given in the following subsections.

##### 4.2.2.1 Waves

Waves have different lengths, heights and directions, and the resulting wave field can have various forms and patterns. Waves create variations in the pixel values in the optical image due to two effects: (a) the wave slopes reflect different amounts of sun and sky light, and (b) breaking waves (whitecaps) create bright patches. Calm-sea situations are usually computationally ideal, as both small and big vessels may be detected with high accuracy. On the other hand, the same vessel detection algorithm that works successfully on a calm sea can give drastically different results when the sea state is complex, especially when detecting smaller vessels. Sea state may range from small, choppy waves caused by wind with wavelengths of only a few decimetres, up to rough seas with waves that may be many tens of meters long. In addition, swell may be present with wavelengths of up to hundreds of meters. These waves are further topped by short-lived waves of centimetre-millimetre wavelengths that constitute the small-scale roughness of the sea surface. For the vessel detection algorithms, the presence of waves becomes a problem when it results in high variation in the background reflectivity (clutter). In optical images, sea surfaces show local texture similarity and local intensity similarity (Yang et al., 2014). The intensity of the clutter is generally proportional to the wind speed and sea state, which means that for high wind speeds and complex sea states it is more difficult to reliably detect all vessels in the area of interest while keeping the number of erroneous detections sufficiently small (Dekker et al., 2013). Also, processing time increases drastically due to the numerous targets to process in the discrimination stage. As such, the algorithms may become ineffective and useless in operational conditions (Jubelin and Khenchaf, 2014). Although an appropriate sea surface analysis can improve the performance of vessel detection and is actually a prerequisite for vessel detection on complex seas, not many algorithms, in fact, take the presence of waves into consideration. False alarms caused by waves are mostly removed in the discrimination phase (e.g. Corbane et al., 2010, Ding et al., 2012, Guang et al., 2011, Zhu et al., 2010). Kanjir et al. (2014) have included a separate algorithm module for sea roughness based on simple statistical descriptions of the sea surface. The roughness scale is divided into 12 levels, which define the level of variability of sea conditions present in the image (the higher the level, the stronger the variability). This roughness level serves later as one of the input parameters for the detection module. To overcome the problem of various surfaces, Yang et al. (2014) applied a method which analyses whether the sea surface is homogeneous or not by using majority intensity number and intensity discrimination degree.

##### 4.2.2.2 Clouds

**Fig. 5:**
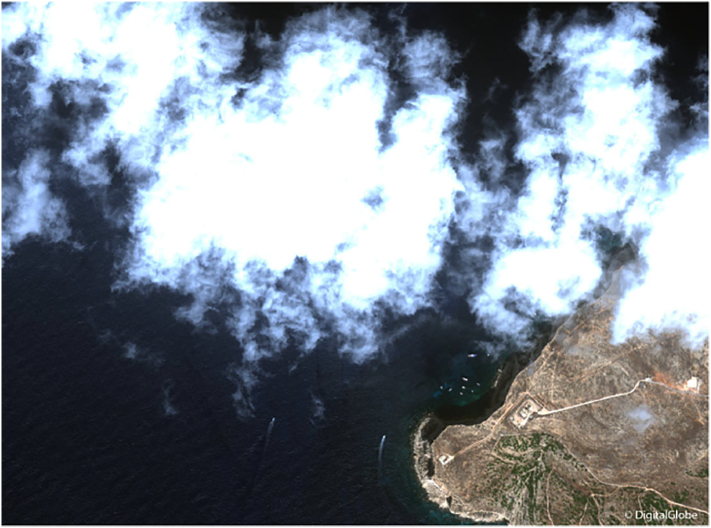
Detail of a WorldView-2 image with a spatial resolution of 1.8 m of the Western region of Lampedusa (Italy), acquired in September 2013 (RGB composite). This image illustrates the difficulty of detecting vessels when much of the scene is obscured by clouds.

Clouds – as true for most applications of optical remote sensing data – negatively affect vessel detection algorithms, and it is therefore important to isolate them (Fig. 5). Full cloud cover obscures the image area in question leading to missed detections, while small, isolated clouds can look like targets, which can only be removed in the classification phase. An objection may be raised that the optical satellite system is inoperative at precisely those times when vessels are at maximum risk (e.g. during storms). However, ship safety and search & rescue is only one application area, and the availability of a position from the constellation in the hours or days prior to an incident is still of potential significance in reducing the uncertainty in location (Bannister and Neyland, 2015).

Some image products are provided with a cloud mask, e.g. the Sentinel-2 Level-1C product which is the one that is distributed (ESA, 2015). Bottom-of-Atmosphere reflectance images are useful as they have clouds masked and haze corrected, but those are not usually available, so more generally Top-of-Atmosphere reflectance products are used. Thresholding techniques are the most used method in remote sensing applications for cloud removal, since clouds have high values in all spectral bands and are therefore relatively easy to eliminate on the basis of the image histogram. Thresholding- or histogram-based methods have helped to remove clouds from the image in the works of Corbane, Pecoul, et al. (2008), Corbane et al., (2010), Daniel et al. (2013) and Kanjir et al. (2014). Further, in the work of Buck et al. (2007) and Jin and Zhang (2015) clouds are removed from the image using a Fourier transform algorithm. This is enabled by the fact that images containing clouds give a sharp rise in the centre of the Fourier transform. The work of Buck et al. (2007) detected any cloud texture, from dense clouds to slight haze. This is also the only work that describes haze elimination on the image. Haze can cause serious inaccuracies, especially during classification since it affects the properties of remote sensing data and modifies the spectral signature of vessels.

##### 4.2.2.3 Sunglint

When satellites observe the Earth in a direction in which the Sun's position causes reflections on the sea surface, a phenomenon called sunglint occurs. In the presence of ocean waves, the sunlight reflected by the ocean wave slopes produces glints on the image according to the relative positions between the sensor, the wave front geometry, and the Sun azimuth and elevation (de Michele et al., 2012). In such circumstances, waves longer than the sensor's resolution can create very high contrasts across their slopes, as some local areas on the waves will be at an angle that reflects the Sun, while others will not. In instances when the sea roughness at small scale (cm up to the sensor's resolution) is low, such contrasts are especially pronounced, whereas under conditions of higher small-scale roughness and weaker long waves, extended areas can become more uniformly very bright. The presence of sunglint or high reflectivity in certain parts of the image can cause a misinterpretation of objects or even larger areas. On images with bad illumination conditions, it is hard even for a human eye to distinguish a target from the background. Researchers that have made efforts to remove this interference from optical satellite data have been mostly dealing with oil spill detection (Grimaldi et al., 2011, Hu, 2011, Liu et al., 2011). None of the works mentioned in Table 2 have dealt directly with sunglint corrections. This is probably due to the absence of sunglint in the test data used for algorithm development. But already a short walk through the Google Earth image viewer proves that this is quite a common effect on the images of water surfaces.

#### 4.2.3 Vessel candidate detection

After the sea-land separation and optional removal of environmental effects, an appropriate vessel candidate detection algorithm has to be applied, presenting the essential part of the whole procedure. The main aim of these vessel candidate detection methods is to successfully indicate pixels that represent possible vessels. Reviewed studies show a wide range of techniques and approaches for isolating the vessel components. It is difficult to categorise them into a mere few groups, especially since proposed approaches are usually composed of a number of sequential procedures (to reduce both the computational time and the number of false alarms, etc.).

For easier readability, we grouped the methods into eight groups as sketched below. These methods are then presented in Table 3 with their corresponding algorithms behind each methodology. In the table we have suggested their advantages and disadvantages and mention only the most relevant works (e.g. articles with SCI index), or works that presented these methods thoroughly and transparently. However, for the details on the proposed algorithms please refer to the original works.

**Table 3:** Candidate detection methods and their positive and negative aspects.

Group of methods	Algorithms	Advantages	Disadvantages	Relevant authors applying methods
Threshold-based methods	Otsu algorithm, histogram-based algorithm, component tree theory, multi-level threshold segmentation	Fast, simple	Good performance on homogenous sea only	McDonnell and Lewis (1978), Buck et al. (2007), Hong et al. (2007), Corbane, Marre et al., (2008), Corbane, Pecoul et al., (2008), Bi et al. (2010), Johansson (2011), Zuo and Kuang (2011), Wang et al. (2011), Zhang et al. (2012), Shuai, Sun, Wu et al. (2016)
Salient-based methods	Itti-Koch model, Jacobs's method, neighbourhood similarity-based method, supervised learning-based saliency model, visual saliency detection method and Chan-Vese model, biquaternion Fourier transform	Relatively good performance on heterogeneous sea	Many false alarms appear if too much clutter is present	Bi et al. (2012), Ding et al. (2012), Guo et al. (2014), Han et al. (2014), Li and Itti (2011), Ma et al. (2010)
Methods based on shape and texture features	Spatio-spectral detector, Mahalanobis distance metric, mathematical morphology, Chan-Vese model, local multiple patterns for texture features, successive shape analysis, hit-or-miss transform, quad tree decomposition	Robust, high detection accuracies	False candidates are still present	Pegler et al., 2003, Pegler et al., 2007, Corbane, Pecoul, et al. (2008), Corbane et al. (2010), Zhu et al. (2010), Huang et al. (2011), Guang et al. (2011), Liu et al. (2014), Harvey et al. (2010), Partsinevelos and Miliaresis (2014)
Statistical methods	Principal component analysis (PCA), Bayesian decision theory	Fast	High knowledge of operator	Wu et al. (2009), Proia and Page (2010)
Transform domain methods	Low and high pass filter, invariant generalised Hough transform, discrete wavelet transform, deep neural in wavelet domain	They weaken the influence of heterogeneous sea	Limited to various complicated sea backgrounds and bright/dark vessels	Burgess (1993), Jubelin and Khenchaf (2014), J. Xu et al. (2014), Tang et al. (2015)
Anomaly detection methods	Intensity discrimination degree, clairvoyant detectors, Reed-Xiaoli algorithm	Robust to strong sea clutter and extreme cases	Poorer performance when dealing with vessels near coast	Yang et al. (2014), Daniel et al. (2013), Shi et al. (2014)
Computer vision methods	Rotating Haar-like feature detector, robust estimator in constant time	Fast, good vessel length estimation	High knowledge of operator	Bouma et al. (2013), Máttyus (2013)
Deep learning methods	Convolutional Neural Network, Stacked Denoising Autoencoder	No need to manually define features	Large training set needed	Tang et al. (2015), R. Zhang et al. (2016), Zou and Shi (2016)

The most commonly used vessel detection techniques are based on the fact that vessels appear brighter than their immediate surroundings; they therefore use local contrast in their detectors (Harvey et al., 2010). The idea of the classical and frequently applied **threshold-based method** is to split an image into parts according to whether each pixel value is above or below a selected threshold value in the histogram. These methods are particularly suitable for the situations with smooth sea surface or high contrast between vessel targets and sea background. **Salient-based methods** are approaches where extractions of salient regions (possible vessels) of an entire scene are calculated and where local features and their interactions across space are examined. These methods have relatively good performance on a heterogeneous sea, but a high increase in false alarms may occur if there is too much clutter on the image. To extract possible vessel candidate regions, some authors take advantage of different characteristics of the vessels and the sea and propose **methods based on shape and texture features,** and usually include also spectral information. These methods are robust and provide relatively high detection accuracies, although false alarm candidates (wakes, clutter) are still present. Various **statistical methods** can also help detect targets on the sea using statistical behaviour of vessels and non-vessel elements in the image. These methods usually yield very fast results, but they also require high knowledge of the operator. The next methods for vessel detection are **transform-domain methods**. They can weaken the influence of varying sea average intensity because they are well adapted to analysing multifractal properties, but can prove to be limited to various complicated sea backgrounds, bright and dark vessels. In these methods, morphological filtering is usually combined to distinguish vessels more easily from the surrounding cluttered waters. We can detect vessels also with **anomaly detection methods** where vessels are represented as irregularities on the surface and can be detected by analysing the normal components of sea surfaces. These methods are robust to strong sea clutter and extreme cases (e.g. dark vessels), but give a poorer performance when dealing with vessels near the coast. The last two groups of vessel detection techniques are both computer science branches and are closely related. In **computer vision methods**, the computers emulate human vision. The methods for object recognition within computer vision are significantly overlapping with the corresponding techniques within remote sensing image analysis. This implies that the basic techniques that are used and developed in these fields are more or less identical. These methods have high performance and provide good length estimation, but at the same time, they require high knowledge of the operator. For the needs of training data, Liu et al. (2017) have compiled HRSC2016 (High Resolution Ship Collection 2016), a set of reference data for vessel recognition from publicly available high-resolution imagery for both at-sea and inshore vessels. The vessel models are organised into a tree structure with three levels (vessel class, category and type). There are 2976 samples for more than 25 vessel classes altogether. The last group, **deep learning methods** are using multi-layer neural networks, and adapt the performance of the method by training algorithms with data. The field of machine learning is concerned with the question of how to construct computer programs that automatically improve with experience (Mitchell, 1997). Here, there is no need to define object features explicitly, but instead, the image data are fed directly to the artificial neural network. While this can be very powerful, a large training set is needed, and if the implementation is not done carefully, new objects that are not well represented in the training set will be misclassified.

For vessel detection, the highest number of authors used methods based on shape and texture (29), threshold-based methods (23) and salient-based methods (23), as these methods tend to be the fastest and simplest. The least number of authors made use of anomaly detection methods (4) or deep learning methods (4). This is to be expected, as the last two methods have been integrated into the remote sensing only recently, with the appearance of images with many details and stronger technology support.

#### 4.2.4 Discrimination methods

The goal of the discrimination is to recognise true vessels among all the detected candidates, i.e. to remove false alarms from the collection of detected vessel candidates from the previous step. The results of the discrimination are objects of two types: vessels and non-vessel objects (i.e. false alarms). Detected non-vessel objects may contain clutter patches, sea wave speckles, islands, and other small noise regions. Since not all the authors use the exact term ‘discrimination’ when performing vessel discrimination, we have resorted to interpreting the process from the paper itself. Detected candidates are usually labelled as ‘vessel’ or ‘other’, but as stated by Bi et al. (2010) sometimes there is no obvious discrimination between the vessel targets and non-vessel ones, therefore false alarms that have the same shape and dimension of a vessel cannot be removed.

Most authors have performed discrimination based on simple geometrical features or their combination. The most calculated geometrical feature for discrimination is the size, i.e. the length and the width of a vessel candidate (Buck et al., 2008, Heiselberg, 2016, Lin et al., 2012, Liu et al., 2014, Liu et al., 2017, Pegler et al., 2003, Willhauck et al., 2005). Also area (Deng et al., 2013, Dong et al., 2013, Partsinevelos and Miliaresis, 2014, Tao et al., 2009) and length-to-width ratio are quite common for discrimination (Chen et al., 2009, Guang et al., 2011, Hong et al., 2007, Jubelin and Khenchaf, 2014, Wang et al., 2011, Xiaoyang et al., 2016). Yang et al. (2014) used compactness as a tool to discriminate detected objects, and a combination of all mentioned geometrical features has been applied in Yang et al. (2015). In all mentioned works, the target is a possible vessel if the variations of each descriptor are below a tolerance parameter.

Some authors focused also on the combination of geometrical features and spectral signatures of the target objects (Burgess, 1993, Corbane et al., 2008, Corbane et al., 2008, Corbane et al., 2010, Dekker et al., 2013, Pegler et al., 2003, Topputo et al., 2015), or they used only spectral features in order to extract possible vessels from the image (Kanjir et al., 2014, Wu et al., 2011).

It is impossible to determine the best approach for discrimination, but given the fact that we are dealing with optical data, the preferred choice is to obtain the results from the combination of geometrical, spectral and textural features of the detected object. The authors mentioned in the last two paragraphs based their results only on discrimination but not classification, so that their final results are isolated, unclassified vessels. The authors listed in the next section have, on the other hand, undertaken also classification, therefore incorporating in most cases discrimination in the classification procedure.

#### 4.2.5 Classification methods

The main aim of the classification is to accurately classify all detected objects of type ‘vessel’ into various classes regarding their characteristics. The classes depend on what is desired and what is possible. They can be simply size-based, e.g. a classification into small, medium and big vessels; they can be fitted to the specific interest, e.g. military or non-military; or they can be detailed, distinguishing between fishing, cargo, tanker, cruise, yacht, tug, dinghy, etc. Due to a similarity of procedures, it is sometimes impossible to draw a clear line between the dichotomous discrimination mentioned in the previous section and classification.

**Fig. 6:**
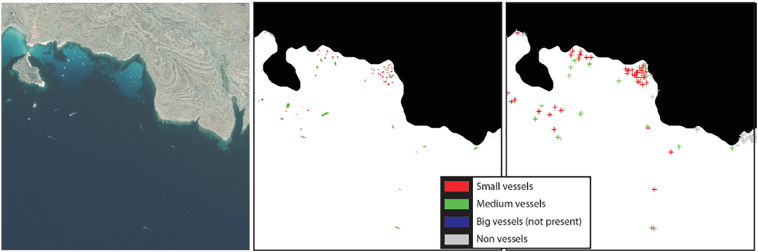
Example of vessel classification on a GeoEye-1 image of Lampedusa (Italy) based on the vessel size. Left: input image. Middle: classified segments with the land removed. Right: classified targets marked with crosses. Small (red) vessels represent detected segments smaller than 20 m, medium (green) between 20 m and 100 m, and big (blue) vessels are the ones measuring more than 100 m. (For interpretation of the references to color in this figure legend, the reader is referred to the web version of this article.)

During vessel candidate detection, segments automatically obtain several attributes, which can be used for classification according to the user's needs. For example, spatial attributes (the size, the ratio of the length to the width, etc.) or spectral characteristics of the vessel targets can be helpful attributes for vessel classification. An example of vessel classification is given in Fig. 6.

The studies used various machine-learning methods to classify detected candidates into the different vessel classes. The most often used method for vessel classification (Table 2) is support vector machine (SVM) – a supervised classification model (Bi et al., 2010, Bi et al., 2012, Guo and Zhu, 2012, Kumar and Selvi, 2011, Li and Itti, 2011, Satyanarayana and Aparna, 2012, Song et al., 2014, Xia et al., 2011). SVM was also adopted as the basic classifier by Zhu et al. (2010) when dealing with the hierarchical vessel classification approach based on the detail pattern analysis. Since many false alarms are common in vessel detection (due to the complicated environmental conditions), the traditional techniques as SVM may have difficulties in efficiently handling such highly varying inputs (Tang et al., 2015).

Some works have shown a shift towards more advanced developments in the field of classification. For example, Corbane, Marre, et al. (2008) and Tang et al. (2015) used a neural network (NN) for high-level feature representation and classification of vessels. Their study demonstrates that this algorithm works fast in terms of detection time and has better generalisation than other traditional learning algorithms.

Other classifiers that were also used for vessel classification were: Bayesian classifier (Antelo et al., 2009), random forest (Johansson, 2011), Fisher classification (Zhang et al., 2012), adaptive boosting algorithm (Shi et al., 2014) and dynamic probability generative model (Guo et al., 2014).

### 4.3 Evaluation criteria for vessel detection and classification performance

With so many independent factors playing a role in detectability, it is not only important to perform detection to a high accuracy, but also to obtain good quantitative estimates thereof. Verification experiments usually suffer from a lack of independent vessel traffic data concurrent with the satellite images, something that can be only partially overcome with fusion of different data. Nevertheless, for users of space-based vessel detection systems, it is important to know, in a given situation, what percentage of vessels may have gone undetected (Greidanus, 2005). What needs to be also considered is which other characteristics of the undetected targets are causing them to remain undetected and what the causes of the false alarms are. None of the analysed techniques from all the reviewed papers was consistently better than all the others, therefore we can state that none is generically optimal, as it is well known that no method is equally good for all images and not all methods are good for a particular type of images. This is due to the different underlying algorithmic approaches (Greidanus, 2005). The challenge, especially when the sea is complex, is the presence of many false alarms, which are hard to eliminate even with visual examination. They present a much higher problem than the false negatives, i.e. the vessels omitted during detection.

Out of all of the examined literature, only 55% have evaluated the performance of their detection methods. To evaluate detection methods, the following numbers and nomenclature are used:

(1) $$Nd_{\mathrm{true}}=\mathrm{No}.\;\text{of correctly detected vessels}=\mathrm{No}.\;\text{of true positives}$$

(2) $$Nd_{\mathrm{false}}=\mathrm{No}.\;\text{of falsely detected vessels}=\mathrm{No}.\;\text{of false alarms}=\mathrm{No}.\;\text{of false positives}$$

(3) $$\mathrm{Nd}=\text{Total}\;\mathrm{no}.\;\text{of detected vessels}=Nd_{\text{true}}+Nd_{\mathrm{false}}$$

(4) Nu=No.of undetected vessels=No.of false negatives

(5) $$\mathrm{Nr}=\mathrm{No}.\;\text{of real vessels}=Nd_{\mathrm{true}}+\mathrm{Nu}$$

With these numbers, most articles use the following performance parameters: detection ratio, missing ratio, false ratio and error figure, or recall and precision. They are defined as:

(6) $$\mathrm{Pd}=\text{Probability of detection}=\text{Detection ratio}=\text{Recall}=\frac{Nd_{\mathrm{true}}}{\mathrm{Nr}}$$

(7) $$\text{Missing ratio}=\frac{\mathrm{Nu}}{\mathrm{Nr}}=1-\mathrm{Pd}$$

(8) $$\text{Precision}=\frac{Nd_{\mathrm{true}}}{\mathrm{Nd}}$$

(9) $$\text{False ratio}=\frac{Nd_{\mathrm{false}}}{\mathrm{Nd}}=1-\text{Precision}$$

(10) Error figure=Missing ratio+False ratio

In addition, maybe the most important parameter is the false alarm rate, Pfa. This is defined as the number of false alarms found, divided by the number of detection decisions taken. If we say that at least 3 pixels are needed for detection (see discussion in Section 5), then the number of detection decisions is the number of pixels that were analysed (N_pix_) divided by 3. So, for a total analysed area of N_pix_ pixels and Nd_false_ false alarms found, the false alarm rate is:

(11) $$\mathrm{Pfa}=\frac{Nd_{\mathrm{false}}}{\left(\;\frac{N_{\mathrm{pix}}}{3}\;\right)}$$

False alarm rates need to be low for most operational applications. Considering e.g. an area of interest of 100 km × 100 km at a pixel size of 10 m, a false alarm rate of 10^−7^ would give 3.3 false alarms, which might be an acceptable number.

In order to evaluate the performance, most of the authors used vessel targets that were identified manually from the image as reference data (Bi et al., 2010, Corbane et al., 2010, Kanjir et al., 2014, Liu et al., 2014). This is usually the consequence of the lack of available AIS data or other reference data in the region. Quite a number of authors compared their algorithms with some other methods (Bi et al., 2012, Ding et al., 2012, Guo et al., 2014, Han et al., 2014, Huang et al., 2011, Li and Itti, 2011, Song et al., 2014, Xia et al., 2011, Yang et al., 2014). An example of a study that deployed targets and collected ground truths is Pegler et al. (2007).

Another criterion also significant in the determination of the performance is time consumption, or how long the algorithm takes to complete the vessel detection and/or classification procedure. With the fast-growing size and number of remote sensing images, the time efficiency of the ship detection algorithm is becoming more and more important, which also brings new challenges to the ship detection tasks (Zou and Shi, 2016). Processing time is especially significant for real-time vessel detection applications, when results need to be delivered quickly. Quite some number of authors listed the time consumption of their algorithms (Bi et al., 2010, Ding et al., 2012, Dong et al., 2013, Guang et al., 2011, Ji-yang et al., 2016, Li et al., 2016, Lin et al., 2012, Tang et al., 2015, Wang et al., 2016, Xiaoyang et al., 2016, Xu and Liu, 2016, Yang et al., 2017, Yang et al., 2014, Yao et al., 2016). The time spent for calculation is strongly dependent on the computer hardware and the results are thus not always comparable.

### 4.4 Data fusion

Optical satellite imagery can be utilised independently or as one of many sources for vessel detection. For a more complete MDA, the data fusion approach, where one combines possible vessel positions from various sources, is suggested. It is important to use as many sources as possible from what is available – AIS, VMS, coastal radar, airborne patrol, SAR data, etc. Most common in the maritime domain is fusion of satellite images and AIS or VMS systems; however, this is mostly done with SAR images (Brekke et al., 2008). Only a few papers dealt with fusion of *optical* and other data (Dekker et al., 2013, Jubelin and Khenchaf, 2014).

Fusion of satellite image data and AIS/VMS entails interpolating or extrapolating the AIS/VMS positions that are available before and after satellite image acquisition time, so as to get the positions *at* the image acquisition time. The accuracy of the interpolated positions is essential for a correct fusion. Minor positional uncertainties between different data sets can occur due to imprecise image georeferencing, but uncertainties can become substantial as a consequence of interpolation if the interval between received AIS/VMS messages is large. As one example of an implemented application, the combination of occasional satellite imagery with continuous AIS/VMS data constitutes an improved control system for unauthorised fishing (e.g. EU, 2009, Art.4.13 and Art.11).

Cooperative systems such as AIS/VMS generally deliver tracks (i.e. series of detections or plots already associated with one another), while non-cooperative EO systems generally deliver single plots from single images (Dekker et al., 2013). With the latter, tracking is much more complicated since there is less information available per vessel to associate detections. To associate plots (detections) obtained at different times to a track, a variety of available information should be exploited – both target attributes (including kinetic, shape, size, colour, etc.) as well as local information such as shipping lanes, etc. (Dekker et al., 2013). Bannister and Neyland (2015) discussed time series of optical images and indicated that, in theory, the combined observations of VHR optical sensors are capable of providing one or more position fixes every day for large vessels (≥ 100 m) and one detection of a smaller (≈ 20 m) vessel every 1–4 days. As to fusing optical and radar imagery, understanding the radar backscatter mechanism and optical reflectance properties is important in selecting appropriate radar wavelengths and polarisations for fusing with optical data (Huang et al., 2010). Jubelin and Khenchaf (2014) are to our knowledge the only researchers that have developed an algorithm for the detection of vessels that works on both optical and SAR imagery of any size in any resolution images. They state that the use of different algorithms for vessel detection for both types of sensors would induce additional costs of development and maintenance as well as difficulties for training and appropriation of systems by operators, and that using a single detection algorithm should limit these difficulties and facilitate the fusion of these two types of detection. However, we believe that this is not necessarily the case, and that dedicated algorithms for optical and radar would always give better results than a single common one used for both, because optical and radar sensors produce intrinsically different results.

None of the analysed papers made use of the fusion of medium or low spatial resolution with high spatial resolution optical images (except for the pansharpening that is done on simultaneously acquired spectral bands). This fusion approach is widely used in other applications to jointly exploit the higher temporal resolution of medium- or low-resolution satellites and the detailed spatial resolution of high-resolution images, but of course depends on the stationarity of the scene.

## 5 Discussion

### 5.1 Current stage of the research

In the past, it was stated many times that far less research has been carried out for vessel detection from optical remote sensing images compared to that carried out for SAR data. Specifically, in 2005, Greidanus (2005) stated that automatic vessel detection in optical imagery is not developed much. Considering the variety of developed methods used in the gathered literature (Table 2), we can state that notable progress has been made since then. Vessel detection using VHR optical images is a hot research topic, covering many different aspects of this field.

#### 5.1.1 Outline of optical satellite sensors

**Fig. 7:**
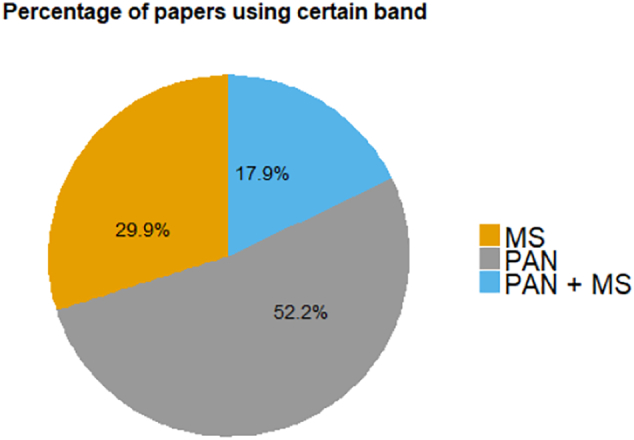
Out of all the authors who defined the imagery used in their paper, the majority (52.2%) used PAN imagery for vessel detection. MS comes second at 29.9%.

As seen in Table 2, around half of the vessel detection methods indicated that their input is only the PAN band, neglecting the MS data, and thus relying more on spatially oriented and less on spectral techniques (35 authors or 52%: Fernandez Arguedas, 2015, Bi et al., 2012, Buck et al., 2007, Corbane et al., 2010, Corbane et al., 2008, Corbane et al., 2008, Guang et al., 2011, Guo and Zhu, 2012, Harvey et al., 2010, Huang et al., 2011, Hung, 2016, Jin and Zhang, 2015, Jubelin and Khenchaf, 2014, Li et al., 2016, Li et al., 2016, Liu et al., 2014, Pan et al., 2015, Proia and Page, 2010, Qi et al., 2015, Shi et al., 2014, Tang et al., 2015, Wang et al., 2005, Willhauck et al., 2005, Xu et al., 2017, Xu and Liu, 2016, Yang et al., 2014, Yang et al., 2015, Yang et al., 2017, Yao et al., 2016, Yokoya and Iwasaki, 2015, Zou and Shi, 2016). On the other hand, 30% (20 authors) use only MS data, and 18% (12 authors) use both PAN and MS data (Fig. 7). This is contrary to the oft-heard argumentation that optical images are used for vessel detection due to the variety of spectral information. This information is apparently used mostly for the classification, since the MS imagery exploits spectral signatures that are possibly unique to the vessel itself (Daniel et al., 2013).

On the PAN imagery, vessels are detected exploiting the total intensity of reflected radiation, whereas on the MS images, vessels are detected either from one explicit band, profiting from specific frequencies of the electromagnetic spectrum, or the combination of many. Kanjir et al. (2014) have used pansharpened images as input data for vessel detection to combine the advantage of the high spatial resolution of the PAN data and the spectral information from the MS bands. They used mostly the NIR band for both sea-land separation and vessel detection, although other MS bands were used for statistical calculations of sea roughness. Also, multi-spectral bands of the 8 m Gaofen-1 sensor were jointly pansharpened to 2 m using the Gram–Schmidt orthogonalisation algorithm in B. Liu et al. (2016). For coarser data, Burgess (1993) concluded that in the Landsat TM imagery, the red and NIR bands are the most useful for vessel detection in marine clear water environments, whereas for turbid water Wu et al. (2009) stated that the SWIR Landsat bands discriminate vessels better than the visible and NIR bands, because these bands are not influenced by turbidity. At the same time, however, the intensity of different vessels in the same band may vary widely because of reflection difference. Some vessels are brighter than their surrounding regions while some are darker than the background in the same bands, depending on the spectral response of a vessel or the effects of its environment. In addition, different parts of the same vessel may have different intensities, which may be caused by different viewing angles and lightning conditions (Ding et al., 2012). Chen et al. (2009) found that vessels are the most distinguishable in the blue band of HJ-1A satellite data. To our knowledge, Ding et al. (2012) is the only publication that conducted an experiment on vessel detection where they handled MS bands as a whole instead of considering them as individual bands. They have shown that handling channels together can provide better results than processing each band separately.

Regarding the resolution, most authors developed their algorithms for vessel detection on images that have a resolution between 0.5 m and 5 m PAN, and around 4 m MS data. This, of course, depends on the year when the algorithm was developed and on the era when certain satellite optical imagery was available. As the resolution of satellite images has been increasing over the years, the tolerable (detectable) minimum size of the vessel should also be taken into account. For example, when 5 m resolution images are used for vessel detection, it is not reasonable to expect to detect vessels smaller than 20 m, as only very limited object details/features can be extracted from a region of a few pixels (Tang et al., 2015). As observed by Topputo et al. (2016), results of vessel detections in simulated 5 m, 10 m and 15 m resolution imagery worsen when spatial resolution decreases; in particular, estimated length values of vessels appear more dispersed. Bannister and Neyland (2015) suggest that detection is possible if the ground sample distance is GSD < *l/3*, where *l* is the vessel length (GSD represents the distance between adjacent pixels centres projected onto the target and is an attribute closely related to spatial resolution). This is consistent with Corbane, Marre et al. (2008) who showed that for a stable analysis, vessels above 14 m in length can be detected from 5 m spatial resolution images.

The majority of authors developed and tested their vessel detection algorithm on two or more optical sensor types, handling many images. Around one third (30%) of the publications tested their algorithm on only one satellite sensor, using only one or a few images. This is understandable for publications written up until the turn of the millennium, where only a few optical sensors were available and access to images was both logistically and financially more demanding. Nevertheless, for higher relevance and wider applicability, it is essential that the proposed algorithm gets to be tested on a representative set of targets and backgrounds (different areas and various meteorological conditions). Ideally, an algorithm would also support a variety of optical sensors. At the same time, we have to take into account the fact that certain methods have been developed and focused on a certain sensor, or similar types of sensors, and are therefore adjusted to its specifications and would not necessarily give good results to images obtained with other optical sensors. It is also important to emphasise that a notable number of algorithms are tested only on selected cropped areas of satellite images, which include the target object but neglect the parts with no targets, which means that the false alarm rates of these algorithms are unverified and may be unacceptably high.

Moreover, most of the papers describe algorithms in development, and only a few authors have developed and discussed their algorithms all the way through to operational implementation.

#### 5.1.2 Outline of vessel detection and classification procedures

As described in Section 4.2.3., a wide range of techniques and approaches have been developed for vessel detection. To better understand the algorithms' performances relative to each other, they need to be applied on the same satellite image dataset, which should be heterogeneous (containing a variety of ship types, acquired under different weather conditions, from various regions of the world, and ideally also acquired by different sensors). Only thus would an adequate comparison of all the methods be ensured and the selection of the best one guaranteed. However, an inter-comparison between the performances of various vessel detection techniques is not an easy task, as each author uses other test data with different settings and targets (small or large vessel, moving vessels, vessels in harbours, general target detection, tracking vessel movements, etc.).

One of the options for sea-land separation is the use of existing GIS data, as discussed in Section 4.2.1. This procedure is fast and easy, but these static water masks also show deficiencies. These are caused by the fact that sea-land masks represent only temporal snapshots of the water bodies distributed worldwide and therefore these masks cannot reflect their dynamic behaviour (Fichtelmann and Borg, 2012). Also, the resolution of existing masks is low and many of the finer details such as man-made structures (e.g. piers) or peninsulas can be left out, resulting in a simplified representation of the coastline (Buck et al., 2008). This problem arises even more when using VHR images. Some of these errors can be corrected manually, but this is time-consuming. The procedures that include separation based on the image itself seem more convenient, since they define the current land-sea separation line (depending on the time of image acquisition) and can be usually performed automatically. Incorrect delineations of the sea and land are mainly caused by turbid water and the presence of uncharted land (e.g. rocks) of similar size as ships. The better the delineation of the land-sea separation, the easier it becomes to detect vessels in the further stages of the analysis.

The same is true when environmental effects (waves, clouds and sunglint) are removed or treated properly on the image. When dealing with highly variant conditions on the sea surface, the processing time might be exponentially increased (Tang et al., 2015). Clouds can be relatively easily eliminated using a histogram threshold to provide a binary cloud–no cloud mask. The problem occurs when there is a haze over the sea surface, modifying the spectral signature of the vessels. Out of all the articles we considered, only one has dealt with this issue (Buck et al., 2007). Although this is a good example of successful haze elimination from a QuickBird image, more approaches concerning this issue in vessel detection applications remain to be developed for other optical sensors.

Waves, varying from smaller whitecaps to an extended pattern of brightness variations of the sea surface, are the main cause of false vessel detections on VHR images. Many of such false alarms are hard to eliminate even with visual examination. We venture to say that many of the vessel detection methods presented in Table 2 would probably not work well on images with complicated sea regions or with other strong environmental effects. The importance of the sea state consideration in vessel detection algorithms is underlined in the work of Guang et al. (2011) and Yang et al. (2014).

Vessel tracks or wakes can be seen in optical images as white foam trails released by the vessel, forming after the propeller. They have linear components and can be detectable even from coarser image resolution (Fisk, 1994). Wakes can be used to estimate vessel heading and speed, and their expanded area can help to indicate the presence of a smaller vessel which otherwise would not be visible on the image (Bouma et al., 2013, Buck et al., 2007).

We should also note that many procedures return the length estimate after vessel detection; some also give the width, the heading estimate or other relevant parameters for the classification. Results in the work of B. Liu et al. (2016) show that most of the ships, whose length-to-width ratio ranges from 3.0 to 7.2, could be identified correctly regardless of their size. But in the case of a moving ship, the size of the vessel segments obtained from optical data is often bigger than the actual vessel size due to its wake. Also Greidanus and Kourti (2006) noted that the separation of a moving vessel and its near wake can be challenging, since they are connected and can have similar brightness, resulting in an overestimation of the vessel size. To perform correct classification, the wake should be separated from the vessel segment in order to estimate the proper vessel length (Dekker et al., 2013); Heiselberg (2016) could successfully do this making use of the NIR band of Sentinel-2.

Around two-third of all the analysed papers (77 articles) have dealt with classification, either with false alarm removal (discrimination) or actual classification into several vessel classes. Fifty-five works have discriminated between vessel target and non-target according to relatively simple attributes or calculations. Simple shape analysis can already remove some obvious false alarms (clouds, large islands, waves). Another 23 works that applied classification to recognise true vessels from the candidates and to classify them accordingly into subclasses have used specific classification algorithms in order to provide different vessel classes. Zhu et al. (2010) have proven that subclasses division and hierarchical classification is helpful for achieving a good classification performance. The same authors also showed that the vessel classification accuracies based on the combined feature sets (attributes) are higher than those based on individual features themselves. Therefore, the combined features are helpful for vessel classification.

There is still much to be done for the classification of small vessels. Vessels less than 10 m in length might still be detected but their classification is almost impossible (Greidanus and Kourti, 2006). It is difficult even for an interpreter to discern e.g. a small fishing vessel from a patrol boat when doing a visual examination. However, it is important to note that vessel classification is desirable, even if not the main objective of the research. Like for detection, there should be a thorough comparison between all mentioned classifiers on the same datasets in order to be able to define the best classifier for final vessel classification implementation.

#### 5.1.3 Evaluation

From all of the collected literature, only two-third (65%) of the publications have performed a quantitative evaluation. This is rather little and disappointing concerning the fact that every developed algorithm should be tested and verified. We could unofficially consider the papers that did not include verification as grey literature. Out of all the works that have performed verification, many authors reported relatively high detection ratios (> 80%). These relatively good results can be discussed from many perspectives. First, a vast majority of the approaches used their own reference data in order to perform the evaluation of the accuracy, and the reference data were mostly obtained by visual interpretation of the authors of the paper who are usually also doing the final test. We would like to suggest with Tomljenovic et al. (2015) that the reference data should be generated by a third party to assure an independent set of data. Second, a majority of works used a small number of images, and mostly the images were taken in a calm sea state. The robustness of the algorithms can only be proven with the usage of heterogeneous images and satellite systems. Last but not least, the detection ratio gives only partial information of the algorithm performance/accuracy. The second important measure is the false alarm rate (ref. Section 4.3). Many false alarms can appear on an image and their discrimination presents a much bigger problem than the missed detections. Therefore, we suggest that when measuring vessel detection algorithm accuracy/performance, apart from considering the detection ratio, it is also of the utmost importance to take into account the false alarm rate.

#### 5.1.4 Data fusion

In the maritime domain, fusion of optical data and other available data usually involves the AIS and the VMS that provide the most frequent observations. Another desired fusion area would be the fusion of optical and SAR data. However, these sensors are not synchronous, and as discussed in Section 4.4, fusion of non-cooperative moving target detections at different times is challenging. Synchronisation between optical and SAR satellite images is difficult for two reasons. In the first place, optical is (near-) nadir looking and SAR is side-looking. SAR imagery starts around 20° off-nadir, but the incidence angles suitable for ship detection are still further out, above 25° and ideally higher, up to maybe 45°–50°. The nadir-looking optical Sentinel-2 reaches an off-nadir angle of only 11° at the edge of its (very wide, 290 km) swath. SPOT-5 could point off-nadir out to 27°, and SPOT-6/7 can to 30°, thereby reaching a marginal overlap with interesting SAR ranges. In its extended mode, SPOT-6/7 can even point out to 45°, but this mode is presumably not routinely accessible and performance (from resolution and atmospheric extinction) is lowered. In the second place, optical satellites tend to pass over around 10:30 local time for optical illumination and cloud conditions, whereas SAR satellites tend to pass over around 06:00 and 18:00 as they prefer dusk-dawn orbits for energy considerations (as an exception, the ALOS satellite SARs pass over closer to noon). So even mounting a SAR (that would need to look at near incidence angles) and an optical camera (that would need to look far off-nadir to image the same area) on the same platform, the quality of the ship imaging of each would be compromised. Also having the SAR and the optical camera on two separate but synchronised platforms is not ideal as the orbits cannot be parallel but will converge somewhere (at the higher latitudes for polar orbits).

### 5.2 Trends and future directions

When taking a closer look at the accumulated methodological approaches of vessel detection and classification from optical satellite imagery presented in Section 4.2, we can observe some trends. Historically starting with the straightforward approach of thresholding bright pixels in a cluttered background, the complexity first increased by taking into account shape, texture and segmentation, and also by applying image transforms to match image content to preconceived shapes. Recently, complexity increased further by exploiting computer vision methods, where the analysis may contain many different steps and is not anymore straightforward at all. The most recent development is that of deep learning, whereby a multi-layer neural network is trained with many samples, which in some aspects makes things easier again because there is no more need to define (or understand) the optimal image features to use for the analysis.

Each of the mentioned techniques implements a specific procedure in detection or classification, but the general problems that most of the research is facing are:

- Complex sea surfaces (sea waves, sunglint, clouds and small islands) give rise to detections as false vessel candidates due to similar characteristics.
- The variation of the reflectivity of vessel targets and/or their components is extremely pronounced, mainly due to the different illumination conditions and the target surface coating materials. This causes vessels to have higher or lower grey levels than the sea surface background (Huang et al., 2011).
- It is difficult to distinguish the vessel from its wake in most optical PAN and MS images.
- Small vessels are hard to classify.
- The general trend to use only a small number of images of only one or a few optical sensors does not contribute to algorithm robustness.

In order to apply ship detection in operational scenarios, it is not acceptable to have to use a different algorithm for each situation (ship type, weather type, image resolution, etc.). On the other hand, it may be a tall order to find a single algorithm that works well in all cases. At least, published vessel detection algorithms should follow a common framework for various optical sensors, and they should be tested on a representative set of sea conditions, ship types, and geographical areas. This would enable them to be generally applicable for monitoring of different maritime activities like commercial fishing, environmental hazards, vessel traffic including traffic pattern analysis, unauthorised migration, and so on. Based on in-depth analysis of all the analysed articles, we propose the following guidelines that studies searching for novel techniques should follow:

- Usage of various optical sensors.
- Verification of the algorithm on the different maritime scenarios.
- Better utilisation of the information contained in the satellite data (use all possible information, e.g. all available bands).
- Improve detection performance especially under difficult conditions.
- Adapt algorithms for classification of (smaller) vessels.
- Comparison of different classifiers to find the best vessel classifier.
- Validation of accuracy, which should include at least detection ratio and false alarm rate.

The quantity of the acquired literature mentioned in this review paper and the variety of the developed methods suggest that vessel detection using VHR optical images is a hot research topic. This trend to develop computational efficient techniques for vessel detection and classification is likely to continue, especially because of the availability of the big amount of free data in the future that will be generated by ESA's Sentinel and other satellites of the latest generation, including small or nanosatellites.

Regarding temporal resolution, the long revisit interval of optical satellite images – multiple days – has been up to now one of the limiting factors in their adoption for operational use in MDA. However, the revisit limitations – which are due to orbit, daylight and clouds – are now being loosened by the launch of large constellations of optical satellites. A web survey executed by the authors in December 2017 reveals a huge increase in the number optical satellites that are already existing or planned for the near future (up to early 2020s): the constellations SkySat, Pléiades-Neo, Scout, WorldView-Legion and OptiSAR promise among them a total of at least 35 satellites at sub-meter resolution; Earth-i, DMC-3, BlackSky and Jilin-1 together foresee some 140 satellites at 1 m resolution; and UrtheDaily and Planet are good for at least 168 satellites at 3–5 m resolution. Some of these will have video capability. When these satellites are better spread throughout the day than the current concentration around 10:30 local time, many updates per hour become in reach. This would start to get sufficient for meaningful tracking of non-cooperative targets. However, this ability would still miss areas of prolonged cloud cover as well as all of the night time, and of course much observation capacity must be trained at the area of interest which may not come at an acceptable cost-benefit ratio.

An alternative approach to enable high revisit frequencies is by geostationary satellite. Many of these are in use for weather monitoring, but they all have resolutions that are much too low for ship detection: the highest existing and planned resolutions are 500 m (COMS-1, Electro M, FengYun-4, GEO-KOMPSAT-2A, GOES, Himawari-8 and -9, MTG-I) or in one case 250 m (GEO-KOMPSAT-2B) (WMO, 2015). However, two have 50 m resolution: the Chinese GaoFen-4 (currently operating) and the Indian GISAT-1 (planned). ESA some years ago entertained ideas for a high-resolution geostationary optical imager (Kunz et al., 2013), but currently none seem to be planned. Such systems may be used ship detection, but how well they perform and how much they can contribute to MDA has yet to be demonstrated.

## 6 Conclusion

This paper attempts to explore and summarise the relevant publications on vessel detection algorithms from various spaceborne optical sensors from 1978 to March 2017. We identified and analysed 119 papers; each one has its own set of procedures and uses its own set of data. The gathered literature allowed us to obtain insight into the current state of the art and to recognise some of the weak and strong points in today's research, thus opening the possibility to predict future trends for this area of remote sensing applications.

The required steps in the whole vessel detection chain from optical images include sea-land separation, vessel candidate detection, elimination of detected non-vessels, and vessel classification; in addition, an optional – yet highly recommended – step is removal or mitigation of environmental effects. An accuracy assessment should also be included when a new method is introduced. The process of isolating sea targets from the background depends on many factors, such as weather, reflections on the waves, solar angle, imaging sensor, the nature of the target and the disturbances it creates. All these factors bring great variations in the selection of suitable methods. Several issues like how to deal with complex sea surface conditions still remain unsolved.

While a lot of work has been done on vessel detection from optical satellite data, the classification of the detected vessels is still underdeveloped. The smallest vessels will always be more difficult to detect and classify than the bigger ones. We presume that exact classification of vessels from optical imagery is the future in the maritime domain awareness development. The final test of any vessel detection or classification method should be its validation on a variety of satellite imagery acquired by HR and VHR sensors, containing a representative set of vessels, under different weather conditions and for various regions of the world. The authors of this paper therefore encourage the organisations in charge to prepare free, open access data sets of optical images, together with ground truth data, for future and existing vessel detection methods to be tested and evaluated.

To have the operational monitoring of vessels depend solely on optical data is not a very attractive proposition, due to the constraints of cloud coverage and daylight availability (night-time imaging is still limited to few satellite sensors and few vessel types). However, in combination with the other surveillance methods like AIS, VMS and SAR, exploiting the multitude of (soon-) available optical satellites can much improve the temporal coverage which is today one of the limiting factors in maritime surveillance away from coastal sensors.

The world of Earth observation is swiftly changing, driven by the availability of open data, the expansion of the small satellite imaging constellations and the rapid advances in digital technologies. We believe that in the near future, remote sensing methods will be adopted as a fully operational monitoring tool in the maritime domain, due to the high amount of data provided by the upcoming commercial constellations and the free and open Sentinel-1 (SAR) and Sentinel-2 (optical) satellites, and the possibility to process the images in near-real time. The new generations of small satellites offer an opportunity to improve the safety and the security of life at sea, especially by improving the coverage of maritime areas. Their increasing capability also offers the possibility of a dedicated electro-optical space-based maritime domain awareness constellation that can realise the full benefit of the concept (Bannister and Neyland, 2015). At last, also the quickly growing field of cloud computing will significantly enhance the data processing capabilities and overcome previously prohibitive processing costs.
